# What Animal Cancers teach us about Human Biology

**DOI:** 10.7150/thno.56623

**Published:** 2021-05-03

**Authors:** Patricia Kattner, Katharina Zeiler, Verena J. Herbener, Katia La Ferla-Brühl, Rebecca Kassubek, Michael Grunert, Timo Burster, Oliver Brühl, Anna Sarah Weber, Hannah Strobel, Georg Karpel-Massler, Sibylle Ott, Alexa Hagedorn, Daniel Tews, Ansgar Schulz, Vikas Prasad, Markus D. Siegelin, Lisa Nonnenmacher, Pamela Fischer-Posovszky, Marc-Eric Halatsch, Klaus-Michael Debatin, Mike-Andrew Westhoff

**Affiliations:** 1 Department of Pediatrics and Adolescent Medicine, University Medical Center Ulm, Ulm, Germany.; 2 Department of Neurosurgery, University Medical Center Ulm, Ulm, Germany.; 3 Laboratorio Analisi Sicilia Catania, Lentini; SR, Italy.; 4 Department of Neurology, University of Ulm, Ulm, Germany.; 5 Department of Nuclear Medicine, German Armed Forces Hospital of Ulm, Ulm, Germany.; 6 Department of Nuclear Medicine, University Medical Center Ulm, Ulm, Germany.; 7 Department of Biology, School of Sciences and Humanities, Nazarbayev University, Nur-Sultan, Kazakhstan Republic.; 8 Department of Pathology & Cell Biology, Columbia University Medical Center, New York, NY, USA.; 9 Animal Research Center, University of Ulm, Ulm, Germany.; 10 Division of Pediatric Endocrinology and Diabetes, Department of Pediatrics and Adolescent Medicine, University Medical Center, Ulm, Germany.

**Keywords:** infectious tumour, transmissible cancer, Peto's paradox, anticancer mechanisms, non-human malignancies, paediatric cancer

## Abstract

Cancers in animals present a large, underutilized reservoir of biomedical information with critical implication for human oncology and medicine in general. Discussing two distinct areas of tumour biology in non-human hosts, we highlight the importance of these findings for our current understanding of cancer, before proposing a coordinated strategy to harvest biomedical information from non-human resources and translate it into a clinical setting.

First, infectious cancers that can be transmitted as allografts between individual hosts, have been identified in four distinct, unrelated groups, dogs, Tasmanian devils, Syrian hamsters and, surprisingly, marine bivalves. These malignancies might hold the key to improving our understanding of the interaction between tumour cell and immune system and, thus, allow us to devise novel treatment strategies that enhance anti-cancer immunosurveillance, as well as suggesting more effective organ and stem cell transplantation strategies. The existence of these malignancies also highlights the need for increased scrutiny when considering the existence of infectious cancers in humans.

Second, it has long been understood that no linear relationship exists between the number of cells within an organism and the cancer incidence rate. To resolve what is known as Peto's Paradox, additional anticancer strategies within different species have to be postulated. These naturally occurring idiosyncrasies to avoid carcinogenesis represent novel potential therapeutic strategies.

## Introduction

**Figure 1:**
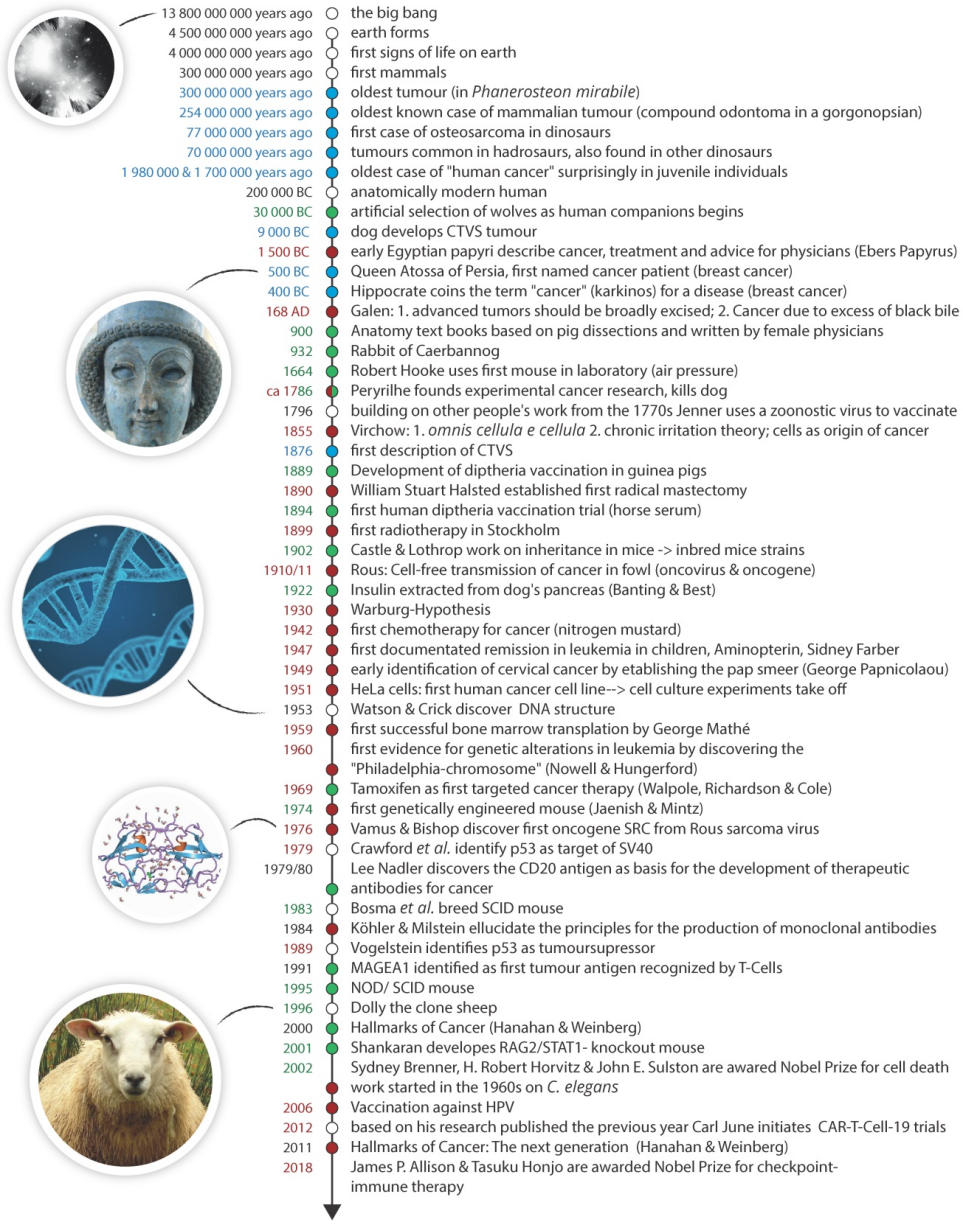
** A brief history of the universe, animal life and cancer.** Shown here is a timeline highlighting the first verified occurrence/naming of novel features relating to cancer (blue), key developments in cancer sciences and oncology (red) and key medical and scientific uses of animals (green).

Data obtained from animals have augmented our medical understanding for more than a millennium. The use of porcine dissections allowed for the creation of early anatomy text books produced around 900 A.D. by (often female) physicians from the Medical School at Salerno 1. This eventually not only formed a basis for modern surgery, but pig cadavers are, for example, still an important part of surgical training in the military 2. Rodent models, while limited in how well they mimic the human situation 3-5, have been an integral and essential part of cancer research for the last century. There are over 450 inbred mouse strains 6 and with the advent of new technologies, such as CRISPR/Cas, creating genetically modified mice has become considerably easier 7. **Figure 1** features selected medical and oncological key events and the contributions from the animal kingdom.

Yet, there remains a certain amount of floccinaucinihilipilification when cancers of non-human origin are discussed outside their uses as surrogate entities, as their medical relevance is often difficult to gauge. However, it is important to note that cancer is also common among non-human animals 8-10 which - with rare exceptions like, for example, the Greenland Shark (*Somniosus microcephalus*) 11 - do not exceed the life expectancy of an average human and - environmental pollutants aside - are less likely to succumb to bad life style choices. Furthermore, there are historical precedences indicating how important zoological cancers can be for oncological researchers. Rous' seminal work identifying the first oncogenic virus in fowl not only led to the discovery of tyrosine kinases and (proto)oncogenes, but preceded (and, therefore, arguably predicted) Epstein's discovery of the first human oncogenic virus by more than 50 years 12.

To highlight the benefits of a concerted approach harvesting data from animal cancers we will use two typical examples from the field of veterinary oncology. First, we will discuss infectious or contagious tumours which have, so far, been identified in four distinct groups of animals and compare them to such entities found associated with humans. Second, we will compare cancer rates in different animals, including humans, and demonstrate how taking into account differences in size, metabolic rates, life span and total cell numbers, we can identify novel, naturally occurring anti-cancer strategies. In the concluding part of this review we will introduce a strategy that will allow for the clinical translation of the previously discussed findings and how the potential benefits will affect several areas of biomedical research and clinical application.

## Cancer as infectious disease

One of the founding moments of experimental cancer research occurred towards the end of the 18^th^ century, when Bernard Peyrilhe injected an emulsion from human breast cancer into a dog, trying to prove the poisonous nature of the disease and its contagiousness 13. With today's scientific understanding, it is not surprising that the dog did not develop a human tumour, but instead had to put down by Peyrilhe's manservant after an abscess formed around the injection site 12. While the experiment therefore can hardly be considered a success (particularly from the dog's point of view) it established not only the use of surrogate animals in cancer research, but also can be seen as a direct predecessor of the work of Francis Peyton Rous, showing the viral origin of some chicken sarcomas 12, which initiated the research into retroviruses as a cause of cancer.

**Figure 2:**
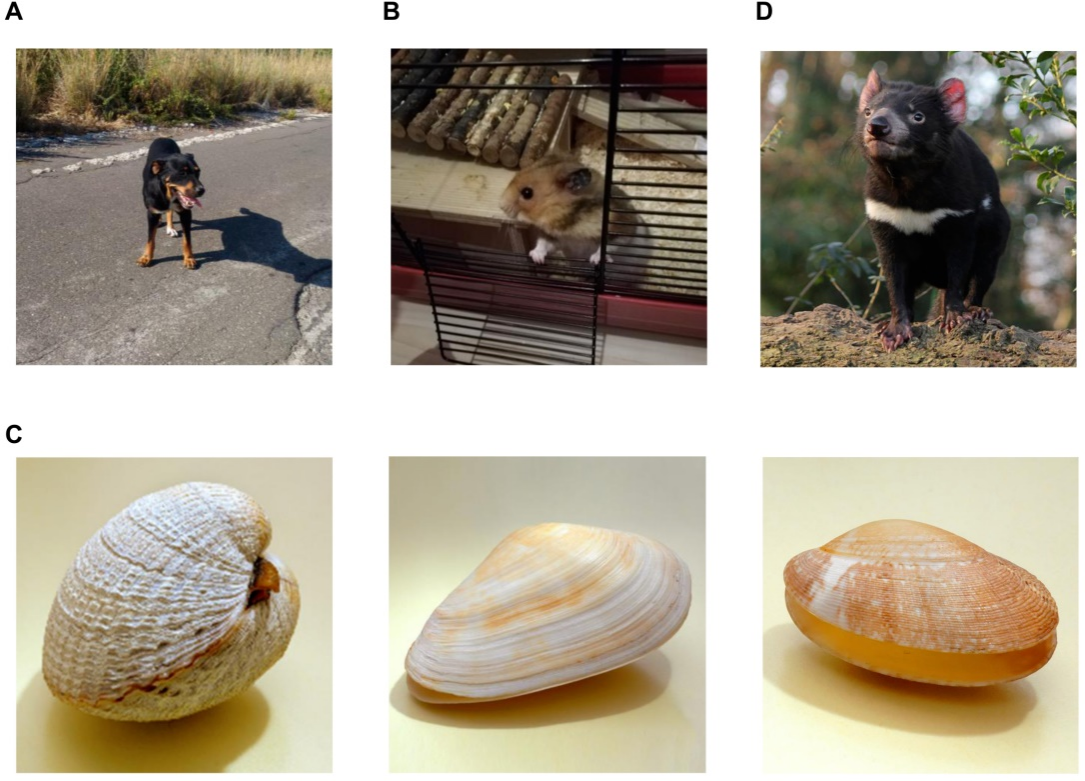
** An unusual quartet of infectious cancer hosts.** Infectious cancer whereby cancer cells can be transmitted between individuals has (so far) been identified in four different animal populations: (A) Dogs (*Canis familiaris*) can suffer from canine transmissible venereal sarcoma (CTVS), also known as canine transmissible venereal tumour (CTVT), transmissible venereal tumour (TVT), Sticker tumour, Sticker's Disease or infectious sarcoma. It is a rare disease in North and Central Europe and North America, where stray dog populations are tightly controlled 19. (B) Syrian (golden) hamsters (*Mesocricetus auratus*) in captivity have been to be affected by contagious reticulum cell sarcoma which apparently can be transmitted between individuals via either cannibalism or a mosquito vector 20, 21. Most of the work on this disease was done during a period of twenty years ending in the mid-1960s 20-22, 24. As no living tumour cells have been preserved it is rather difficult to assess these findings with modern analytical tools 25. (C) Commercially probably the most interesting transmissible cancer was affecting bivalve molluscs was first identified in the 1970s and has caused a steep global decline in bivalves 26. The leukaemia-like disease has so far been identified in soft-shell clams, mussels, cockles and golden carpet shell clams at both sides of the Atlantic Ocean, as well as in the North Pacific Ocean 26, 27. Left to right: The common cockle (*Cerastoderma edule*), the soft-shell clam or sand gaper *Mya arenaria*, the pullet carpet shell (*Venerupis corrugate*). (D) The best-known transmissible tumour which also received extensive coverage in the popular press is doubtlessly the devil facial tumour disease (DFTD) in Tasmanian devils (*Sarcophilus harrisii*) 28-32. First noticed in 1996, the disease had spread to more than half of the species' range by 2007 and by 2008 affected populations had been reduced by 89% 28.

Interestingly, a similar experimental set-up as used by Rous, of filtering extracts through a fine mesh to ensure a cell- and bacteria-free solution, had previously failed when applied to a canine tumour 14, the canine transmissible venereal sarcoma (CTVS) or Sticker's Disease 15. This infectious cancer, which was later shown to be able to cross major histocompatibility barriers and even infect related species, like wolf, coyote and fox 16, was ostensibly not of viral origin. 130 years after its initial description the cause of CTVS was discovered: the tumour cells themselves are infectious, i.e. live tumour cells can be transplanted, while exposure to neither dead cells nor cell-free filtrates will result in a tumour 17. These tumours are generally transmitted between individuals during sexual intercourse; infection is followed by four to six months of an initial growth phase (P phase), a short stable phase and frequently a regression phase (R phase) upon activation of the host's immune response 18. Metastasis is rare, occurring in approximately 5% of all cases and, unless occurring to the central nervous system or eye, does not affect the overall good prognosis of total remission 19. While surgery is considered rather ineffective, CTVS responds favourably to chemotherapy and radiotherapy 19. There are only a few reported cases of clonally transmissible or parasitic cancers, where malignant cells behave, in essence, as allograft (**Figure 2**). CTVS aside, there are to date only three other parasitic cancers identified, a leukaemia-like disease in bivalves, contagious reticulum cell sarcoma in hamsters and devil facial tumour disease (DFTD) in Tasmanian devils. It is the latter which has probably been most reported on outside the medical literature, as it drove the host species to the brink of extinction 28. DFTD tumour cells are transmitted via biting, be it during mating rituals or fighting, and form an ulcerating tumour around the mouth and go on to destroy the jaw bone eventually interfering with feeding. No effective cure for this disease has been suggested so far, however, recently receptor tyrosine kinases which are already targeted in human cancer therapy have been identified as putative target for DFTD treatment 29, while immunotherapy using MHC class I-expressing tumour cells as a vaccine also showed great promise 30. Additionally, it has also been reported that populations of Tasmanian devil might be rapidly evolving immune-modulating resistance to this infectious tumour 31.

Unlike DFTD and CTVS, contagious reticulum cell sarcoma in hamsters has so far been described only under experimental conditions in a series of papers from the early to mid-1960s 18-22, 24. Originally presenting as a highly metastatic large ulcerating tumour, at the terminal phase the peripheral blood was flooded with tumour cells 20, later publications only refer to its leukemic manifestation 21. Based on an earlier observation, that hamsters readily form spontaneous sarcomas that are experimentally transmissible 23, it was shown that contact exposure between transfected and uninoculated individuals was sufficient to cause cancer transmission 20. This was initially assumed to be due to the cannibalistic nature of hamsters, but subsequently demonstrated also to be possible due to transmission via a vector, the *Aëdes aegypti* mosquito 21. Interestingly, it would appear that the malignant cells were experimentally transfected, which caused changes in tumour behaviour, prior to the contact experiments 20. It is therefore unclear whether the original, spontaneously arisen, reticulum cell sarcoma was already contagious. In addition, this contagious sarcoma also differs from the cells causing DFTD and those initiating CTVS, as the latter are of clonal origin, while the transmissible sarcomas in hamsters have been suggested to have spontaneously occurred on several occasions 25. It is also unclear, due to the limited possibilities in the 1960s, whether, despite initial reports, a virus might not have played a role in tumour initiation or transmission after all 25. This issue is unlikely to be successfully resolved, as cells from all transmissible tumours in hamsters are no longer available 25.

Finally, a fourth transmissible cancer was identified in marine bivalves, such as clams and mussels. Apparently, a leukaemia-like disease, - molluscs only possess one circulatory fluid called haemolymph - it was first identified in the 1970s and has spread through various populations and caused a drastic global reduction in bivalves 26. Originally, soft-shell clams (*Mya arenaria*) from Main, New York and Prince Edward Island were found to be infected with identical cancer clones 26. Further analyses identified additional infectious leukaemia in mussels (*Mytilus trossulus*), cockles (*Cerastoderma edule*) and golden carpet shell clams (*Polititapes aureus*) from diverse locations, such as North-American west coast and the seas at Galicia (Spain) 27. These findings are rather surprising, as marine bivalves are generally not the most mobile creatures, being sessile in adulthood. While DFTD and CTVS are transmitted via direct contact, the route of transmission is unclear in marine bivalves. The most likely scenario suggests that cancer cells are taken up via filter feeding, i.e. free-floating leukaemia cells are present in contaminated sea water 26. While this would suggest that incredibly low numbers of cells are sufficient to seed a leukaemia in a new host, it does fit with the observation that cancer cells from soft-shell clams are able to survive more than six hours in sea water 33.

Contagious reticulum cell sarcoma in hamsters and DFTD in Tasmanian devils have in common that they affect a population with little genetic diversity. All Syrian or golden hamsters (*Mesocricetus auratus*) are descendants of a single female, presumably one of the critters originally captured in Aleppo in the 1930s 23, 34, while the population of Tasmanian devils was already declining prior to the advent of European settlers 35. Furthermore, DFTD was first described in 1996 and spread rapidly to an endemic distribution, and in addition, the appearance of multiple strains and the high lethality, all suggest an evolving parasite that is badly adapted to its host 25. In contrast, CTVS is believed to have arisen 11,000 years ago and started to spread widely through the canine population 500 years ago 36. It is, therefore, the oldest and longest continuously cultured (in an *in vivo* host) cell line in existence.

Despite their surprising abundance in marine bivalves, transmissible tumours are a rare occurrence. In 5,500 species of mammals only three types have been proposed to exist (in the case of transmissible sarcoma in hamsters, there still seems to be some uncertainty), there have been - so far - no reports of transmissible cancer in non-mammalian vertebrates which make up some 55,000 described species 25. However, as seen with the DFTD, there is no reason to assume that new forms of transmissible cancer cannot arise spontaneously in the future.

### Speculations on transmissible cancer in humans

What then is the situation in *Homo sapiens*? The first data on the potential transmission of cancer cells between individuals comes from a series of experiments by Chester M. Southam, which can only be termed highly unethical or rather criminal. Investigating the potential mechanisms by which the immune system kills tumour cells, or “the natural killing off process of the human body” 37, Southam was responsible for live cancer cells (among them HeLa cells) being injected into 14 terminally ill cancer patients and over a hundred^A^ prison “volunteers” 37-39. He was later also involved in similar experiments in 1962 on 22 senile patients at the Jewish Chronic Disease Hospital in New York City, again displaying a rather flexible attitude to informed patients' consent by telling the patients they would receive human cells grown in a test tube to avoid “the phobia and ignorance that surrounds the word cancer” 37, 40, 41. Although these experiments preceded the National Research Act of 1974, which was - at least in part - created as a response to the similarly unethical Tuskegee syphilis study, the Nuremberg Code already postulated voluntary, well-informed, understanding consent in 1947. When three M.D.s refused to participate in the 1962 experiment and went public^B^, Southam had his license revoked for one year. Two years later he was elected president of the American Cancer Society 41. In addition, there are also less substantiated reports of 300 healthy women being injected with HeLa cells at the Sloan-Kettering Institute. As this is the same institute Southam worked at, these experimentations are usually implied to be also associated with him 40, 42. It has been reported that the physicians involved were aware at the time that their treatment might cause cancer 40.

Despite these large cohorts of subjects, the actual data from these experiments is difficult to ascertain. Southam seems to suggest that subcutaneous implantation of cancer cells (even of animal origin) led to the formation of nodules after approximately a week post-injection and that cells were cleared from the body within 4 weeks 38. In both populations, terminal cancer patients and prisoners, non-cancerous cells did not grow well locally and while, even after a follow-up of five months, prisoners experienced no difficulties, four cancer patients experienced recurrence after the implanted cancer cells should have been removed and in one case metastasis to the axillary nodes occurred 38.

The fatal danger inherent in such research was laid bare by another ethically dubious study where the fatal melanoma of a 50-year old woman was transplanted for 24 days to her 80-year old mother 43. The mother developed disseminating metastatic cancer 86 days after transplantation, but (surprisingly) large, toxic doses of phenylalanine mustard led to an apparently complete recovery. However, the patient died 451 days after transplantation, her body riddled with metastases 43.

While not explicitly stated in the actual papers, the implication of these data sets seems to follow Paul Ehrlich's dictum from 1909, namely the immune system recognizes and protects from most arising cancers 44, i.e. the cancer cells were eliminated by the immune system of the healthy prisoners, while the cancer patients were too ill to mount an effective immune response. In the case of the mother/daughter transplant we might be dealing with similar, HLA haploidentical serotypes and an aging immune system in the 80-year old woman. This also fits with the described cases of naturally occurring infectious cancers where cancer is passed on from the mother to her unborn child. While it had long been speculated that this is a (rare) possibility for melanoma or haemopoietic malignancies, it was only proven in 2009 when a leukemic clone in an infant was positively identified as of maternal origin 45. Similar transmissions are also believed to have occurred between twinned foetuses *in utero* 46.

The most unusual case report of transmissible tumours involves both animals and humans. An HIV patient with a history of non-compliance was found to have apparently malignant cells in lymph nodes and lungs, however, their small size suggested a non-human origin 47. The cells were identified as originating from *Hymenolepis nana*, the most common human tapeworm, which is not known to develop cancer. Furthermore, a genetic analysis revealed mutations in the tapeworm analogues of human genes associated with cancer 47. It is most likely that the microenvironment provided by the host allowed the development of malignant tapeworm cells which then crossed the species barrier, making this not only an infectious cancer, but also a zoonotic disease. From the perspective of cancer researchers this case appears rather unique and the explanation of the underlying molecular changes rather speculative, however, responses from zoologist showed less scepticism, citing similar cases dating as far back as 1905 48.

### Reasons for the low prevalence of infectious tumours in humans

There is no *a priori* biological reason, therefore, why infectious cancers cannot arise, or indeed have not arisen in the past in humans. While the current data suggest that a healthy immune system offers some protection, it does not appear absolute. CTVT and DFTD are both transmitted during mating. While we like to think human courting rituals are rather distinct from other mammalian species^C^, this is objectively not the case: the world wide prevalence of cervical human papillomavirus (HPV) infection is 10% 49, 36.7 million people were infected with human immunodeficiency virus (HIV) in 2015 50 and it has been estimated that almost 1 million people per day become infected with either chlamydia, gonorrhoea, syphilis or trichomoniasis 51. Interestingly, both HPV and HIV are, of course, associated with an increased cancer risk. While a viral infection can be already transmitted during an occult phase, a sexually transmissible tumour needs to be of a certain size to be transferred between individuals, i.e. the non-affected participant can clearly identify the affected organ. This is, however, unlike to provide an effective barrier to transmission: Unfortunately, non-consensual or monetary-based sexual intercourse where a partner (usually the female) has no option to decline are still far too common. There are also plenty of examples where the presence of a sexual transmitted disease was the initiator of intercourse; the virgin cleansing myth, for example, were the rape of the most vulnerable members of society is believed to cure diseases like AIDS and syphilis 52.

In addition, biting during feeding and cannibalistic behaviour, aspects which has been proposed to spread transmissible cancer in Tasmanian devils and hamsters respectively, are also uncommon among *Homo sapiens*. However, how uncommon cannibalistic behaviour has been throughout human (pre)history is a hotly debated topic 53-55 and there are certainly cultures where cannibalism has occurred in the absence of additional stressors, such as famine. The best studied example of which are the Fore of Papua New Guinea who practiced, and according to some reports still practice, a form of mortuary cannibalism 56. Interestingly, this ritualistic behaviour has also been associated with a disease, kuru, which has been proposed to be a prion disease that is transmitted by eating the brain of deceased family members 57.

While the presence of opposable thumbs also leads to a feeding ritual very distinct from that found in most other animals, a factor probably equal or more important than any *mores* or societal taboos that prevented the potential spread of parasitic cancer via cannibalism among humans, is our species' 1.5-million-year-old association with fire and cooking 58. Cooking and roasting followed by exposure to stomach acid surely would function as a potent barrier for infectious cancer cells, although in the case of the Fore does not protect them from a prion disease 56^D^. Analogous, the handling of raw, uncooked meat can also suffice to cause viral infections. If current hypotheses regarding the species jump of SIV (simian immunodeficiency virus) to HIV are correct, then the emergence of this infection is also due to almost cannibalistic events, the slaughter of our closest living relative, chimpanzees (and also gorillas and mangabeys), for bushmeat 61. While SIV can infect humans in a laboratory setting it does not cause an overt disease 62, so to cause a pandemic SIV had to first evolve mechanism to avoid the immune system, tetherin in particular 61. These mechanisms are remarkably similar to what has to occur for tumour cells to become transmissible.

### A clinical caveat: Transplants as source of transmissible cancer in humans

While organs and donors are screened prior to transplantation, there is a surprisingly high rate of occult cancer present. It has been reported that 0.04% of organ and about 0.06% of hematopoietic stem cell transplants contract cancer 46. This corresponds to around one third of all recipients of organs donated by people with occult cancer developing cancer 63. Whether the remaining two thirds develop no cancer just due to the fact that no malignant cells are in the transplant, or whether their bodies reject the cancer cells remains to be elucidated. Some observations suggest that the latter cause can at least play a contributing role 46. However, with an estimated annual 126,670 organ transplants worldwide (estimate for 2015 by www.statista.com) this would suggest more than 50 cases of infectious cancer in humans per year. The actual numbers could be higher, if one considers that organ transplants also carry a 1 in 200 risk of leading to Kaposi sarcoma, a disease generally believed to be caused by the presence of human herpesvirus 8 (HHV8) in immunocompromised individuals 46. Recipients of organ transplants might develop Kaposi sarcoma via three distinct routes: either the organ is already infected with HHV8 and the immunosuppressed environment of the donor mediates viral spread, or the donor is already HHV8 positive and the immunosuppressive medication needed for the transplant allows viral reactivation, or the transplant organ already contains neoplastic cells 64. Barozzi found the presence of donor-specific markers, i.e. identifiers for a transmissible tumour, in five out of eight Kaposi sarcomas which developed after organ transplantation 64. Therefore, infectious or contagious cancer has also been clearly identified in humans, admittedly under artificial, highly specific circumstances, and should be considered an important contributor to human health issues.

Finally, there are also a few cases of transmissible tumours in humans which cannot be explained by a close relationship between recipient and donor or by immunosuppression of the host. Among the most comprehensibly studied are the accidental transfer of a sarcoma from patient to surgeon, where both had different HLA haplotypes with complete discrepancies of *DRB1* and *DQB1* alleles 65 and the accidental injection of human colonic adenocarcinoma cell line into a healthy 19-year-old laboratory worker, where an allograft occurred also despite HLA mismatches 66.

## Novel protection mechanisms against cancer

While the precise molecular mechanisms and details are still being controversially discussed, it is generally accepted that (adult) cancer is primarily a stochastic disease. There is a tight correlation between the number of (stem) cells and cell divisions in a particular organ and the likelihood of developing cancer in said organ 67, 68. Therefore, a thought experiment could easily lead to a conclusion that - *ceteris paribus* - compared to humans, a creature with more cells which need to be produced by cell division, or a creature with longer life span leading to a higher turnover of cells should be more susceptible to cancer. Or restated: long-lived animals need to be considerably smaller than humans while larger animals can only be short-lived before succumbing to cancer. This is clearly not the case as the aforementioned Greenland Shark has the longest known lifespan of all vertebrate species and is also one of the largest living species of shark, growing up to 6 meters in length 69. If one concentrates on mammals only, mathematical modelling suggests that - again *ceteris paribus* - 96% of all elephants should develop cancer, while the likelihood of a blue whale not developing cancer throughout its life should be less than 1 in 2.27×10^53^ 70^E^. While several recent studies have also found no correlation between cancer rates and body size between mammalian species 70-72, this observation was originally made more than 40 years ago by Peto and colleagues 73, 74. Peto calculated that humans having 1000-times more cells and living 30-times longer than mice human DNA would need to be a billion or a trillion times more “cancerproofed” than murine DNA to allow for equal cancer risk 74. He concluded that this is rather unlikely to be the case and postulates that some evolutionary considerations must therefore account for the fact that humans do not die at an early age of multiple carcinoma 74. The actual cancer rate in laboratory mice is difficult to determine, as different strains exhibit a huge variation in spontaneous cancers 75, 76, but a recent study looking at 9,219 mice across 13 strains found that 0.9% of mice exhibited tumours upon necropsy 77. Assuming a relative cancer risk in humans of 1.0 (see Table 1), then the relative risk of a wild mouse can be calculated (based on average weight and size and also taking the metabolic rate into account) as 1.45238738218312 × 10^-8 F^. This would imply that, if no additional difference between mouse and man existed than, even in the absence of human behaviour that increases cancer risk (it is, after all, fairly safe to assume that most mice a non-smokers and do not excessively sun bath without appropriate protection) 61,966,938.78% of all humans should be positive for cancer upon their death. This discrepancy between observed fact and mathematical prediction, which we have summarized for several mammalian species in Table 1, is generally referred to as Peto's Paradox and several solutions to this conundrum have been put forward. There are three models that attempt, in general, to explain these observations:

### The metabolic rate hypothesis

A side effect of the high energy demand/increased metabolic rate of transforming cells is the concurrent increase in the production of reactive oxygen species (ROS). ROS, in turn, are known mutagens, inducing DNA replication stress and play a critical role in carcinogenesis 81, which further supports the notion of an important metabolic contribution to carcinogenesis 82.

Based on these observations the so-called metabolic rate hypothesis tries to combine the known effect of metabolism, including ROS-production, with the observation that larger mammals develop less cancer than one would predict. In contrast to death due to tumour incidences, the basal metabolic rate is correlated to body size. This is known as Kleiber's law, which postulates that an animal's metabolic rate increases to the power of 0.75 in relation to its mass 83. The difference in mass between human and mouse is 2,800-fold 84, but the difference in metabolic rate is only 2,100-fold (2,800×0.75), i.e. humans need to produce less watt per gram tissue than a normal mouse. Therefore, larger animals have a lower basal metabolic rate and, thus, produce lower amounts of ROS, resulting in a decreased oxidative stress level and hence a lower frequency of cancer 85, 86. This argument is augmented by additional supporting parameters, e.g. the levels of protein carbonyls and the activity of glutathione peroxidase as markers of oxidative stress or the absence thereof 87.

As metabolic turnover also affects genetic expression patterns through epigenetics, Takemoto and co-workers combined in an advanced study the metabolic rate hypothesis and the gene-abundance-hypothesis which postulates that bigger animals have a higher number of tumour suppressor genes and, therefore, a lower incidence rate of cancer 88. Correcting the correlation between number of genes and body size by taking into account the mass-specific metabolic rate, they only found a significant increase of immune-system related genes with increasing body mass and concluded that the gene-abundance-hypothesis is more related to metabolism than to cancer 88. Interestingly, this seems to contradict findings with regards to the elephant, which we will discuss below.

While there are several studies supporting the metabolic rate hypothesis, different theoretic approaches regarding the physiology of the hypothesis are used. For example, the WBE (West, Brown and Enquist)-Model is based upon nutrient delivery as key factor of the lower basal metabolic rate 89, while other studies, not without controversy, postulate a multi-factorial model 90, 91. There are also authors who argue that a low basal metabolic turnover is not the only factor influencing the physiology of Peto's paradox. Maciak and Michalak, for example, postulate that the size of cells in relation to body mass is an underestimated factor and, therefore, concluded that slowly dividing cells with a lower basal metabolic rate are the underlying physiology of the paradox 92. Of course, one also needs to consider cellular aging over time.

Other studies, however, contradict the metabolic rate hypothesis: Costantini and co-workers analysed markers of oxidative stress in Greenland sharks 93. As mentioned, they are very long-lived and even if they are not mammals, as vertebrates they are still similar enough to be worth considering. In contrast to other recent studies with long-lived species, the authors found signs of high oxidative stress and high antioxidant levels. Following the metabolic rate hypothesis, one would expect low rates of antioxidants. There was also no correlation with the measured data and maximum lifespan. Hence the authors assumed that environmental conditions like hypoxia and deep diving would be a more likely explanation for the longevity of Greenland sharks 93.

### Different endogenous retroviral load

While looking into the relationship between retroviruses and their host genomes in relation to immune responses, Katzourakis and co-workers made the interesting discovery that the genome of mice has accumulated much more small ERVs over the last 10 million years, than the genome of humans 94. ERVs, or endogenous retroviruses, are endogenous viral elements most likely derived from retroviruses.

Retroviruses which have been around for about 100 million years contain a single-stranded positive-sense RNA molecule but form a DNA intermediate that integrates into the host's genome. If they infect germ cells and are transmitted from one generation to the next, thus becoming fixed in the host's gene pool, these elements are considered ERVs. As ERVs have a tendency to accumulate mutations more easily (because they do not code for proteins that are necessary for the cell's survival), they do not necessarily remain infectious. Around 1% to 8% of the human genome is believed to consist of ERVs 95, 96. Retroviruses are known causes of cancer: the first oncogenic virus, Rous Sarcoma Virus, is a retrovirus that had 'captured' a host gene (the tyrosine-kinase Src) by integrating close to it and incorporating a permanent active, truncated version into its viral genome 12, 97. Aside from making imperfect copies of the host's proto-oncogenes and turning them into viral oncogenes, retroviruses can also facilitate cancer formation by inserting into and, thus, inactivating tumour suppressor genes.

Based on their initial observation, Katzourakis and co-workers tested a total of thirty-eight mammalian genomes, identifying more than 27,000 unique viral sequences. Their findings suggest that small mammals have more ERVs in their genome than larger mammals and, therefore, they conclude that bigger and long-lived animals protect even more efficiently against ERVs 94.

However, at least in humans, ERVs are not considered a major cause of cancer, although the mechanisms that prevent integration in larger animals could also be associated with a more general protection of genomic integrity. Intriguingly, while immunocompromised mice do not necessarily exhibit higher rates of spontaneous cancers than other laboratory strains, the NOD-*scid*/*scid* mouse is more susceptible to spontaneous thymic lymphoma, which appears to be due to the endogenous ecotropic murine leukaemia provirus locus 100, suggesting that ERVs at least can play a role in strain-specific cancer susceptibility.

Another aspect well worth studying is the prevalence of cancer-associated retroviruses, as opposed to ERVs, in different species. For example, Rous sarcoma virus and Avian leukosis virus in fowl, Mouse mammary tumour virus and Murine leukaemia virus in mouse, Feline leukaemia virus and Feline immunodeficiency virus in cats, the Bovine leukaemia virus in cows and Jaagsiekte sheep retrovirus in sheep, to name but a few which have at least an economic impact in husbandry. To our knowledge the different impact of the viral prevalence in different species on the overall cancer susceptibility of these classes of animals has never been systematically studied.

### Hypertumours

Peto's original observation concentrated on mice and men and later studies attempting to confirm his observation concentrated on domestic or zoo animals 70-74. Data on wild animals, especially big mammals, is rather limited and one has to be careful not to draw conclusions based on very limited numbers of individuals. Attempts to expand the existing data sets - for example with the California sea lion (*Zalophus californianus*) 101 - have not really caught on. Most cases of cancer in wildlife are only examined sporadically, as with the cancer incidence in cetacean populations. The available data suggest that malignant transformations in cetaceans are very rare with thirty-three documented cases worldwide 9. Interestingly, in one population of beluga whales living in the St. Lawrence estuary higher numbers of cancers were found in the stranded members of the population, reaching rates comparable to humans 9. However, this seems to be a unique case and has been linked to the agricultural and industrial pollution of the estuary 9. Nevertheless, considering their weight of up to 1,500kg and a length of 2.6 to 6.7 meters 102 and a maximal life expectancy of sixty years 103, one would expect much higher cancer rates than in humans.

Focusing on large mammals and cetaceans in particular, Nagy and colleagues offer a largely hypothetical model that might explain why big animals do not die of cancer sooner/more often 70. There is a significant difference of tumour type and malignancy in small mammals with lower body mass compared to mammals with higher body mass and longevity. Generally speaking, small creatures die more frequently from any type of tumour, highly aggressive to less malignant, as a small tumour mass is already sufficient to kill the host. The difference between large and small animals is primarily numbers of cells and total cell divisions, while size of a particular cell type does not vary greatly between species 84^G^. In humans, for example, a tumour is lethal, on average with large variations depending on type and localization, when it has acquired a mass of approximately 1 kg, consisting of 1x10^12^ to 1x10^13^ cells 105. An average human weighs approximately 70 kg, i.e. the cancer accounts for around 1.4% of the total body weight. A similar tumour in a beluga whale would account for 0.07% of total body weight and, thus, very likely affect the host very differently. For a tumour to account for a similar percentage of total body weight in beluga, it would have to weigh 20 kg (and be composed of roughly 2×10^13^ to 2×10^14^ cells). Nagy and colleagues postulate that this increased potential would be sufficient to create an environmental niche complex enough to lead to the formation of a hypertumour. In essence the tumour might grow complex enough to be itself afflicted by cancer 70. A hypertumour is, therefore, defined as a highly aggressive secondary tumour that is growing on the primary tumour, presenting itself as necrotic regions associated with histological and genetic markers of aggressive growth 70. In contrast, in small mammals hypertumours do not have time to evolve because the host is already dying from (the size of) the primary tumour.

The idea of a hypertumour was first raised in 2004 when Nagy modelled tumour behaviour as a function of an ecological community 106. He later expanded the model to identify two causes that could lead to the development of a hypertumour within the original tumour: insufficient angiogenesis leading to the 'hijacking' of existing vascular infrastructures within the tumour by cells that themselves produce insufficient amounts of tumour angiogenic factors 70, and an increased demand for phosphorus, leading to an up-regulation of phosphate transport proteins and, thus, upregulated ribosome biogenesis 107. While the hypertumour model certainly solves some problems raised by Peto's paradox - and indeed, cancer cells have been identified that compete with other populations of malignant cells 105 - one should bear in mind that tumour size, i.e. the amount of abnormal cells present within a growth, is not necessarily a determinant of aggressiveness/deadliness, i.e. small tumours can kill via metastasis and tissue destruction resulting from invasion, while benign tumours can reach an incredible mass without being lethal.

In addition to the three general mechanisms proposed, there must be other contributing factors. Some of these have been described for individual species.

As mentioned above, were there no differences in anticancer mechanisms between human and elephant, 96% of all elephants should develop cancer 70. As this is clearly not the case, *TP53* is a tempting candidate to investigate and indeed, the African elephant (*Loxodonta africana*) has 20 copies of the gene 72.*TP53* “is arguably the most important tumour suppressor“ and its gene product, p53, is involved in almost all key aspects of cell behaviour that define the hallmarks of cancer 114, 115, such as cell-cycle arrest, apoptosis, DNA repair, senescence, anti-angiogenesis, autophagy and metabolism antioxidant 116. It is, therefore, not surprising that it is found mutated in more than 50% all of cancers, varying from less than 5% in cervical cancer to 90% in ovarian cancer 116. Inactivation of p53, which is a common initiating mutation in many types of cancer, is harder to achieve in elephants, as more copies have to be mutated to inactivate the protein. To the extra copies of p53 probably contribute to elephantine lymphocytes being more prone to DNA damage-induced apoptosis than their human counterparts 72. It is possible that this is not the only anticancer strategy to be found in elephants. In addition, novel transcriptional targets of p53 have also been identified in elephants, such as the functionalized leukaemia inhibitory factor pseudogene (LIF6), the gene product of which translocates to the mitochondria and induces cell death upon DNA damage. LIF6 is transcriptionally upregulated by p53 117.

The naked mole rat (*Heterocephalus glaber*), for example, has several identified mechanisms that reduce its risk of developing cancer. This long-lived creature displays some fascinating traits, such as being eusocial and lacking the ability to sustain thermogenesis 118. They also live in complete darkness and under low oxygen conditions, but while exposed to high levels of oxidative stress, they display no signs of oxidative damage and are therefore considered “a challenge to the theories that link ageing, cancer and redox homeostasis” 118. With a life span exceeding 30 years naked mole rats are the longest-lived rodents. For comparison the similarly sized house mouse has a life expectancy of around four years 119. While initially presumed to be resistant to spontaneous, as well as experimentally induced cancers 118, oncological growths have now been identified in at least two members of this species 120^H^. Nevertheless, in contrast to mice, where more than 55% exhibit cancerous lesions upon necropsy 121, these creatures are highly resistant to cancer and several mechanisms have been proposed to mediate this.

As seen with the elephant example above, p53 has also been implicated in mediating cancer resistance in the naked mole rat 122. Specifically, naked mole rat p53 has been described to have a more than ten times longer half-life with a larger proportion of the protein being localized in the nucleus even prior to DNA damage when compared to mouse and human p53 121. Transcriptional regulator activities as well as tumour suppressor functions are retained in this p53 variant 121 suggesting a priming of the cells to DNA damage responses 123. In addition, fibroblasts from naked mole rats were shown to be hypersensitive to contact inhibition of growth, activating - via p53 and pRb - p16^Ink4a^. In contrast, in human and mouse fibroblasts (and naked mole rat fibroblasts in the absence of early p16^Ink4a^ activation) contact inhibition is mediated by p27^Kip1^ at a much higher density 122. Early contact inhibition appears to be mediated by naked mole rat fibroblasts secreting elevated levels of a high molecular weight hyaluronan five times larger than human or mouse hyaluronan 119. In addition, these creatures have an extra, fourth splice variant transcribed from the *INK4a/b* locus 124. This splice variant, pALT^Ink4a/b^ is induced by various stresses and by cell contact and - even when expressed in human cells - is a more potent inducer of cell cycle arrest than p16^Ink4a^ or p15^Ink4ab^ 124. If one imagines tumour development as an orderly multistep progression model 125, this hypersensitivity should already affect hyperproliferation, i.e. dysplasia formation and, therefore, stop tumour formation prior to malignant transformation. While it is tempting to speculate that this particular mechanism originally evolved to provide the naked mole rat skin with increased elasticity needed for living in underground tunnels and was later co-opted into an anti-cancer mechanism 119, clearly more research is needed.

As not all tumours progress in such an orderly fashion, one would expect additional anti-cancer mechanisms in an organism which has never been afflicted with a tumour in the wild 119. Indeed, it was also reported that naked mole rat fibroblasts have an unusually structured 28S ribosomal RNA, which might contribute to the higher fidelity in translation, while maintaining a similar translation rate as found in mice 126. This would indicate that naked mole rats produce fewer aberrant proteins, and while the influence on cancer formation was not directly tested, it fits the observation that these creatures have a surprisingly stable proteome 118.

Interestingly, most anti-cancer research in the naked mole rat has focused on fibroblasts and at this stage one can only speculate what other anti-cancer mechanisms might be revealed in other cell types, particularly the tissue stem cells.

Another critter which uses high molecular weight hyaluronan is the blind mole rate (*Spalax ehrenbergi*), which - despite the name - is more closely related to mice and rats than to the naked mole rat 127. Also, unlike the fibroblasts in the naked mole rate, the cells of the blind mole rat do not display early contact inhibition and it is believed that here the hyaluronan in combination with low activity of heparanase contribute to a more structured extracellular matrix 127. In addition, cells from the blind mole rat display a rather unique form of cell death, termed concerted cell death, which seems to result from a massive release of IFNβ upon rapid cell proliferation; here, the aim is not to eliminate to hyperproliferating cells, but to destroy a large area that contains those cells - Seluanov and colleagues compare this to a scorched earth strategy 127.

With expression profiles and sequencing efforts becoming more common, further information on other species are to be expected. In bats, for example, miRNAs have been identified to be differently expressed to other mammals, alterations in growth hormone signalling and DNA repair mechanisms were also highlighted, that might explain the relative resistance of long-lived bats to cancer 127. We have highlighted some additional, potentially interesting animal tumours not discussed here in **Table A.**

## Discussion

Cancer as a disease has afflicted our species long before the emergence of modern humans approximately 200,000 years ago 140. New archaeological evidence suggests that neoplastic growths were not rare events in human ancestors, with the earliest *hominin* cancers identified being 1.7 and 1.98 million years old and, interestingly, associated with juvenile individuals [141, 142, respectively]. These findings already suggest that, while it is true that the cancer burden surged over the last century 143, it is an oversimplification to assume that these malignancies are just the results of increased control over other life-threatening diseases, extended average population age and detrimental cultural influences, such as smoking 144. This is also reflected in the first appearances of cancer in animal species (the oldest tumour was identified in a 300 000 000-years old specimen, the oldest mammalian tumour is 254 000 000 years old, see **Figure 1**). How interconnected human and non-human medicine are, was also experienced by one of the authors who, as a paediatrician, ended up treating a new-born orangutan at the University Children's Hospital, avowedly not for cancer 145.

**Figure 3:**
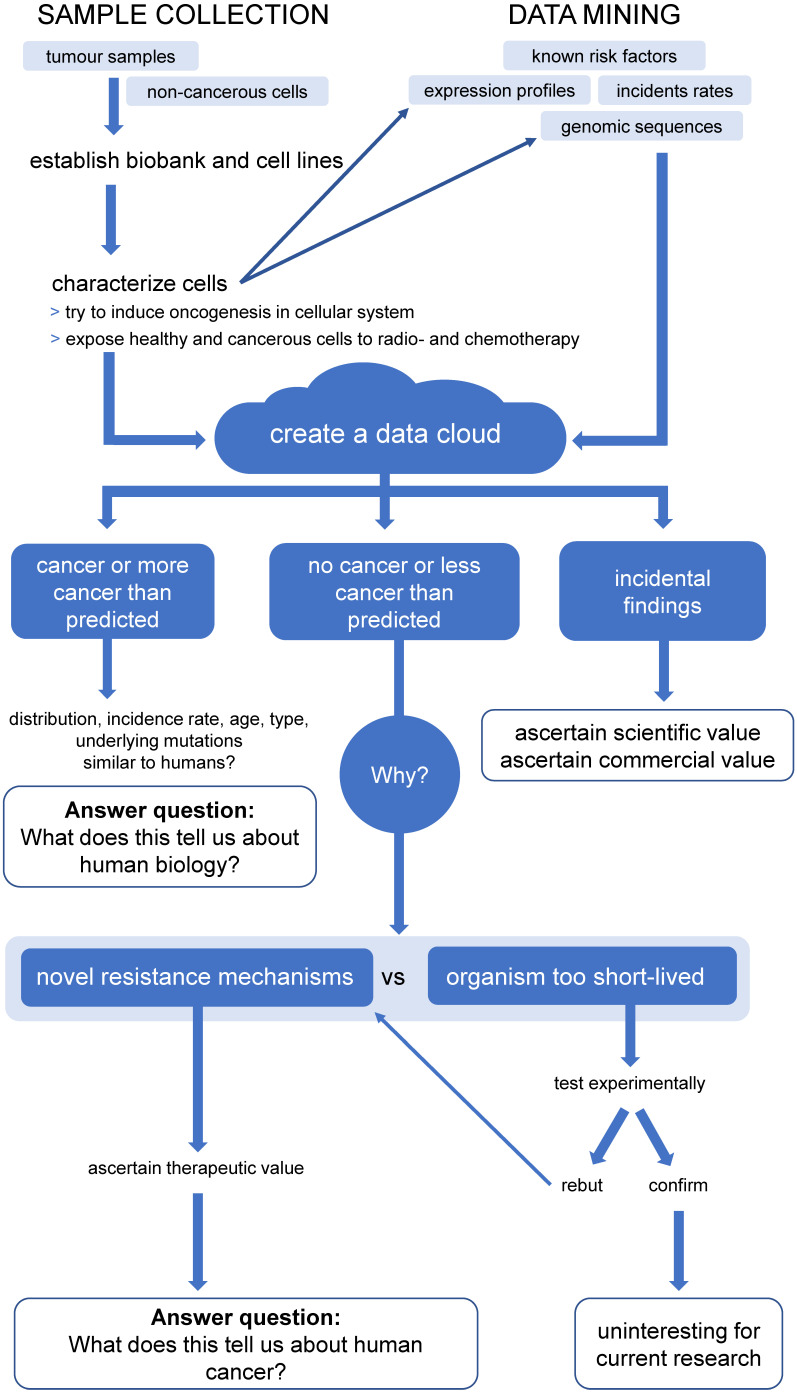
** Proposed workflow for the Animal Cancer Cloud (ACC), a potential project to harvest biomedical information from animal cancers.** Proposed workflow of a project combining establishing a biobank of animal tumour samples, across a wide variety of species and tumour sites, including healthy controls and a data mining approach.

Yet, despite their long parallel history, animal tumours have, so far, not been efficiently used as the potent research tool that they are. Summarised in **Figure 3,** we propose a project to harvest potential biomedical information from animal cancers. Here, we have depicted the proposed workflow of a project that combines establishing a biobank of animal tumour samples, across a wide variety of species and tumour sites (including healthy controls) and a data mining approach.

Central to our proposal is the creation of a data cloud collecting and collating information on non-human cancers, as well as animal genomes. While this is doubtlessly a daunting multi-generational proposition, combining the efforts of The Human Genome Project and The Cancer Genome Atlas for up to all 7.77 million animal species suggested to exist 146, many data sets already exist or are currently being produced. For example, The Darwin Tree of Life project proposes to sequence the genomes of all 70,000 species of eukaryotic organisms in Britain and Ireland (https://www.darwintreeoflife.org/). Led by the Wellcome Sanger Institute this cooperative effort has already attracted considerable funding. Furthermore, several mammalian genomes have already been sequenced, so that, with relatively little effort of sequencing the corresponding mammalian cancers, the feasibility of an Animal Cancer Cloud (ACC) could be demonstrated.

We predict three major lines of knowledge will emerge from the ACC: 1) the distribution of different kind of cancers in different animals. Comparing and contrasting this information will help us to better understand human biology, not limited to cancers. 2) Identifying novel cancer resistance and susceptibility mechanisms and their clinical potential. 3) Historically, working with animals has yielded considerable incidental findings, which have been both of scientific and commercial value. It is therefore conceivable that the ACC will also produces such findings, which might partially help further fund this project. There are at least three key areas where information harvested from the ACC approach will provide an important contribution.

### Are animal cancers better surrogates for paediatric tumours?

Recent molecular findings suggest that human paediatric cancer should be viewed as disease group distinct from environmental factors- and aging-induced cancer in adults 147, 148. Cancer predisposing germline mutations can be detected in 5 to 10% of all patients with childhood cancers and while adult tumours display multiple genomic alterations, e.g. polyploidy and multiple chromosomal aberrations, paediatric tumours have a low mutational load and exhibit only a few genetic alterations 149.

**Figure 4:**
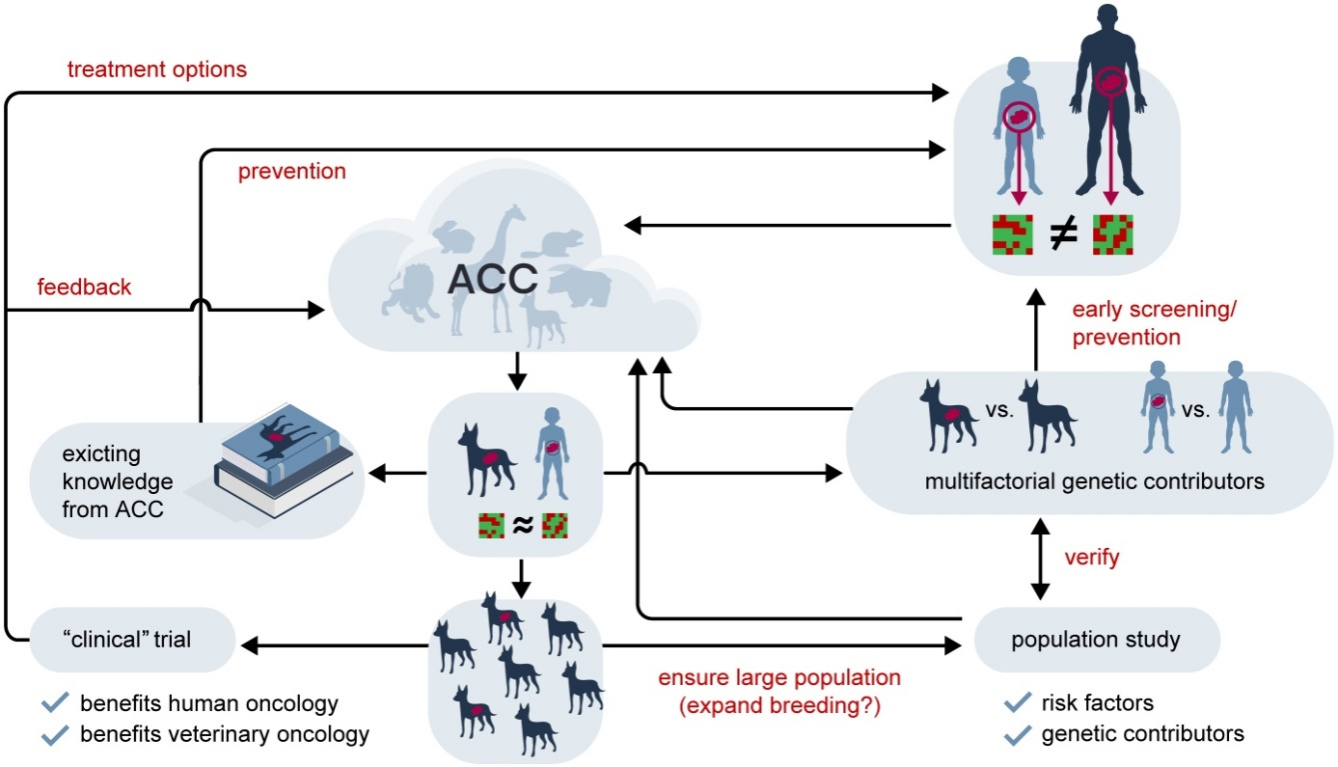
** The potential future value of the Animal Cancer Cloud in the treatment of rare cancers.** Rare human tumours which are too distinct in their (epi)genetic characteristics to allow the application of findings from common tumours might have analogues in animal cancers. Shown here as an example is a generic paediatric tumour compared to an adult disease, but this line of reasoning holds true for rare adult tumours, such as, for example, a Juxtaglomerular cell tumour, as well. Using the data accumulated in the Animal Cancer Cloud (ACC) an animal homologue with similar characteristics might be identified. If this is the case historical records might yield information beneficial for humans with regards to potential risk factors and treatment strategies. Information from the animal population might also be used in concert with human genetics to identify multifactorial genetic contributors to this cancer type, potentially leading to an early screening/prevention strategy in the at-risk human population. If the selected a) animal fulfils certain prerequisites, such as short life span and small body size and a large enough population exists or can be bred and b) the tumour is common enough or can be induced a clinical animal trial can be envisioned, where treatment options are evaluated in a genetically diverse population with the disease in its natural environment. It is expected that such trials would be superior to the traditional pre-clinical *in vivo* models and would allow a more efficient preselection for the clinical evaluation in humans. They would, of course, also create more data for the ACC. Finally, a population-based study would also produce additional information on associated risks for the animal tumour which could also contribute to our understanding of its human counterpart.

The time to cancer development in many (shorter lived) species is closer to that of paediatric cancers than of adult humans. **Table 2** compares common cancers in different mammals and human children and adults, indicating that the difference between species is by no means larger than between differently aged humans. For example, lymphoma are among the three most common cancers in cat^I^, dog, horse, rabbit and human child, but not human adult (Non-Hodgkin lymphoma is the sixth most common cancer in adult). From the kinetic point of view the time to tumour is much more similar in cat, dog, horse, rabbit and human child compared to human adult and child. In all animals considered we have also reduced genetic variation through breeding, so we might gain considerable knowledge from studying zoological malignancies that we can transfer to paediatric malignancies. While no one would argue that studying adult and paediatric cancer does not reveal informative similarities, looking outside our own species, we rarely acknowledge that information might have potential implications for human medicine. This is of particular importance, as in terms of research material available and potential candidates for clinical trials, paediatric tumours must be considered rare and while animal cell lines and pre-clinical evaluation in an appropriately matched non-human population cannot replace the need for paediatric clinical trials, they might contribute to their reduction by earlier identification of unpromising candidates. A potential, future workflow addressing these issues is outlined in **Figure 4.**

### What do infectious cancers tell us about the immune system and how does that affect transplant medicine?

One of the basic questions to be asked with regard to “infectious” cancer is: Why are the cells not recognized as foreign? The answer is complex, but almost certainly involves the major histocompatibility complex (MHC) which is central to the vertebrate immune system (**Table B**).

**Figure 5:**
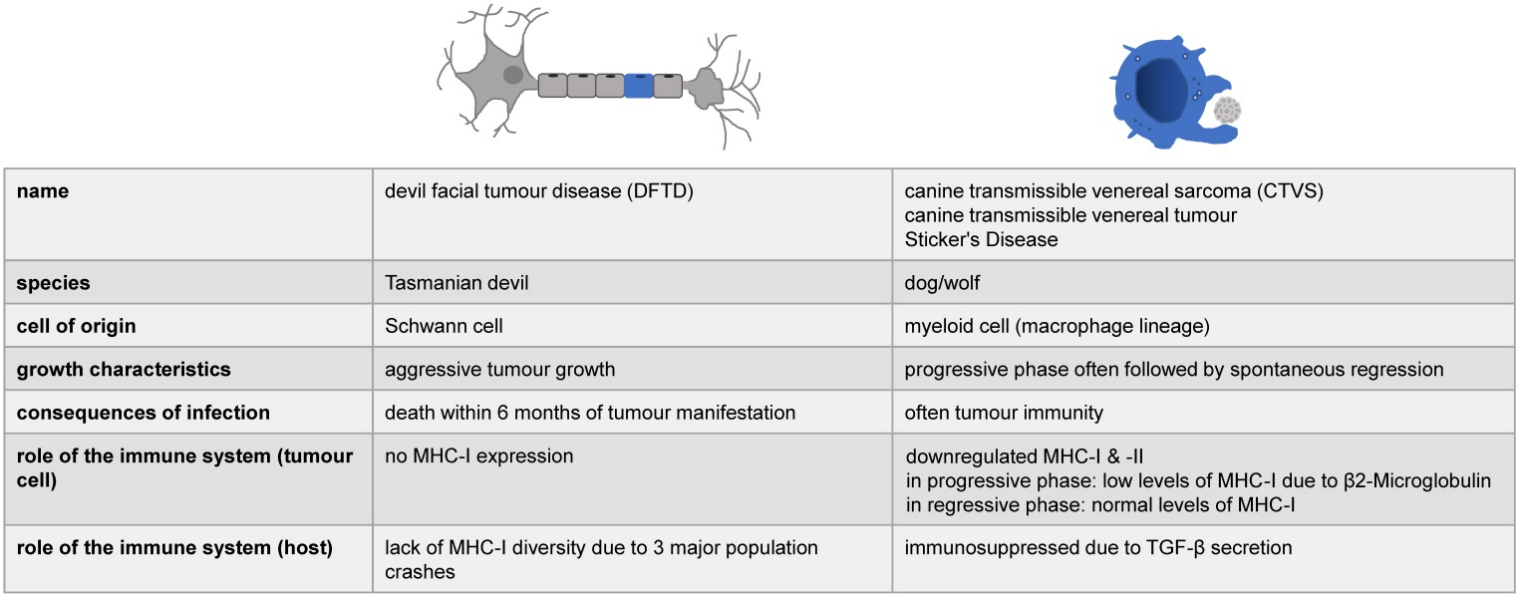
** The immune system and infectious cancers.** The role of the immune system has so far been studied in two of the four known forms of infectious cancer. Here, we have summarized the spares, partially contradictory information available.

No data exists for the mechanisms involving molluscs and hamsters, but for Tasmanian devils and dogs, it was shown that the cancer cells possess mechanisms by which they reduce the host's immune response (25, see also **Figure 5**). In the case of CTVS it was initially proposed that the tumour cells downregulate MHC class I expression, while MHC class II molecules were virtually absent 17, however, a Gene Set Enrichment Analysis showed that virtually all genes involved in immune surveillance harboured partially redundant mutations 25. This actually further complicates matters, as CTVS is the only infectious cancer with a reported rate of spontaneous regression, i.e. a potential re-activation of the immune system might occur that fights off the infection 25. CTVS cells were also found to secrete toxic molecule(s) that specifically kill peripheral blood B lymphocytes 18. In addition to the lack of genetic diversity in Tasmanian devils, DFTD also displays no MHC class I on its cell surface 25. In the case of an *in utero* transfer of leukaemia from (human) mother to offspring, a deletion of HLA alleles in these tumour cells was observed, which were not inherited by the infant and would be otherwise foreign for the immune system, suggesting a possible mechanism of immune evasion 45. Therefore, infectious cancers provide an interesting model in studying the immune system and provide potentially important mechanistic models of how to improve transplantation/reduce tissue rejection, while reducing graft-versus-host reactions and the need for immunosuppressive medication.

Many molecular interactions also play a role in graft rejection, but allogeneic differences of the class I and II loci are the most important. Organs transplanted between MHC-identical individuals, such as identical twins, are readily accepted. However, organs transplanted between MHC antigen-mismatched individuals are rejected without immunosuppressive therapy. The HLA polymorphism is thus an important immunological barrier in the transplantation of solid organs and the risk of acute/chronic rejection due to incompatible HLA antigens persists. That is, the better the recipient/donor HLA compatibility, the better the chances of successful organ transplantation 154.

Haemopoietic stem-cell transplantation is a powerful therapy in the treatment of high-risk haematological malignant disorders and other life-threatening haematological and genetic diseases. The main complication is graft-versus-host disease (GvHD), which affects many organ systems, e.g. gastrointestinal tract, skin and lungs. Thus, many transplant recipients must be treated with immunosuppressive drugs, which may include the increasing risks for serious infections 155.

While over a million patients suffering from malignant or non-malignant diseases received hematopoietic stem cell transplantation (HSCT), due to the morbidity that comes along with the treatment of GvHD, HSCT is still very limited in its applicability 156. GvHD is a common complication after stem cell transplantation where T-lymphocytes, which remain in the donated tissue, recognize the cells of the recipient as foreign, attack them and causes infectious complications and organ failures. A follow-up study of HSCT recipients shows that long term survivors (more than 20 years) had an eight-fold higher risk to develop new malignancies, including solid tumours, hematologic malignancies and post-transplant lymphoproliferative disorder, than the non-transplanted population 157. This is most likely due to a complex interplay of DNA damage due to conditioning, inborn cancer susceptibility and other factors. However, it has also been suggested that intense immunosuppression and in most of the cases the proliferation of Epstein Barr Virus (EBV) after stem-cell and organ transplantation contribute to this 158-160. The relationship between immunosuppressive therapy and malignancy after transplantation has been demonstrated by epidemiological data. Duration of exposure to immunosuppressive therapy and intensity play an important role in the risk of malignancies following transplantation. Furthermore, a more aggressive tumour progression with accelerated growth and metastasis under advanced suppression therapy has been described, which is associated with a lower survival rate of patients. Indirectly, immunosuppressive drugs significantly increase post-transplant malignancy risk by facilitating the oncogenic virus effect. Initial reports of reduced incidence of cancer among organ transplant recipients receiving treatment with mTOR inhibitors strongly suggest separate pathways for pharmacological immunosuppression and oncogenesis 161.

Currently there is no method that suppresses the host's immune response to antigens of the graft while maintaining other immune responses. Therefore, rejection must be prevented by nonspecific immunosuppressants. However, these drugs interfere with specific and nonspecific immunity in transplant recipients and thus increase the risk of contracting an infection and/or malignancy 162. Usually immunosuppressive drugs from the drug groups calcineurin inhibitors, antiproliferative agents, antibodies and glucocorticoids are used to prevent a rejection reaction. The underlying mechanisms of infectious cancers might indicate an approach that would allow transplants in the absence of immunosuppressive drugs, thus increasing overall health of the patients and - ironically - reducing the risks of future malignancies.

### Novel cancer resistance mechanisms and their role in human medicine

While the validity of Peto's Paradox is not universally accepted 163, it nevertheless remains an important question whether the anti-cancer methods evolved in other mammalians might benefit *Homo sapiens*, both in general terms and specifically. For example, ultra-high molecular weight hyaluronan, one of the proposed mediators of cancer resistance in the naked mole rat, has been suggested as the basis of an anti-cancer nanoparticle therapy 164, while the findings in elephants might benefit the sufferers of a rare genetic disorder.

Li-Fraumeni syndrome (LFS) is a hereditary cancer syndrome, which might affect 1 in 20,000 to 1 in 50,000 individuals 165, 166. LFS increases the risk of developing cancer - mainly sarcoma and tumours in the breasts, brain and adrenal glands - by the age of 30 to 50%, compared to 1% in the general population, and 90% by the age of 70 167. It is generally associated with a germline mutation of the *TP53* gene, encoding the p53 tumour suppressor, which is inherited in an autosomal dominant fashion^J^. *TP53* is also found to be mutated in 50% or more of all cancers 116. It is therefore tempting to envision a therapeutic approach based on the data gleaned from elephants that introduces multiple copies of *TP53* into the human genome and thus lowers the overall risk, both of LFS patients and in theory the general population of cancer^K^. Interestingly, when creating mutant mice lines which have increased p53 activity, which would mimic the effects of several copies of *TP53*, this led to premature ageing of the mice 170. While this suggests that ageing might be a price to be paid for tumour suppression 171, it leads to a new perplexity as to why elephants with multiple copies of TP53 can reach an age of over eighty years 172.

Seluanov and colleagues have in a recent Opinion article making a convincing argument for utilizing our understanding of how long-lived mammals avoid oncogenesis to create small molecule mimics of those anti-cancer adaptions and use them in a clinical setting 127. For instance, one can easily envision the increase in quality of life in patients with familial adenomatous polyposis or somatic *BRCA1* and *2* mutations where localized treatment leads to increased p16^Ink4a^ activity, as seen in the naked mole rat, and thus prevents tumour formation. Even when current treatment is considered highly effective, such as in the case for many paediatric malignancies, it is often associated with severe long-term side effects, such as dementia, cognitive decline, hearing loss and hypothalamic-pituitary dysfunction associated with radiotherapy after brain tumour treatment, as well as the emergence of secondary malignancies associated with radio- and chemotherapy 173-176. Here too, a more gentle, targeted approach based on molecules found in certain animals might lead to a more persistent improvement in the quality of life.

In addition, highlighted as incidental findings in our proposal, we predict that novel information of scientific and commercial interests will also emerge from enhanced research into animal cancers. For example, hibernating animals, such as bears, might hold the key to reducing negative side effects of obesity in humans and potentially reveal new treatment approaches for diabetes 177. While a recent genomic analysis of the blowhead whale, considered the longest-lived mammal, revealed not only the expected changes of expression in genes associated with cancer and aging, but also demonstrated that whales express a truncated form of uncoupling protein 1 (UCP1) 178. UCP1 is a mitochondrial carrier protein and is specifically expressed in brown adipose tissue. In the inner mitochondrial membrane, UCP1 enhances proton conductance which leads the uncoupling of the oxidative phosphorylation from ATP production and to dissipation of the proton motive force as heat 179. While Brown adipose tissue is only present in mammals, giving them an evolutionary advantage, orthologs of UCP1 can be also found in other taxa including amphibia, fish, tunicates, insects and even plants, where UCP1 presumably plays another role apart from uncoupling 180. Interestingly, the naked mole rat also expresses an unusual form of this protein 118. Another incidental finding in sharks and camels (and possibly a third group, shark-like fish) revealed the existence of small, but functional antibodies that lack the light chains 181. These antibodies were further developed into so-called Nanobodies which are currently not any being clinically evaluated for the treatment of various diseases, including cancer, but also used diagnostically and were essential in determining the crystal structure of the β2 adrenergic receptor-Gs protein complex 182. This work was awarded the Nobel Prize in Chemistry in 2012.

Finally, while outside the scope of this paper, it should be pointed out that increased rates of cancer in animals which will be detected by the ACC can also serve as an indicator and, thus, a monitor for environmental pollution, as has been reported for beluga whales from the St. Lawrence estuary 9, a California sea lion population on the west coast of the USA 101 and Hawaiian Green Sea Turtles 183.

In summary, a concentrated effort to map and study non-human cancers has the potential not only to further our understanding of human malignancies and biology, but also has the potential for additional promising scientific and commercial ventures. Two recent publication further support our proposal to establish a systematic research network in this area. Hernández and co-workers passionately argue for increased use of comparative oncology between human and veterinary clinics in the context of targeted therapy 184, while the team around Michael Metzger, who were the first to describe infectious cancer in molluscs, outline the value of bivalves as models for human health 185. With recent large-scale sequencing techniques and data handling becoming both more automated and more affordable, as well as several projects already producing important data sets, we will not only argue that a concerted effort to produce the ACC is feasible, but also highly promising from both a scientific and commercial point of view.

## Footnotes

**A:** While this range is quoted in some secondary publications, the original study only mentions that “[f]rom a large group of volunteers, 14 were chosen for the initial study“ 38, in contrast Skloot quotes a number of 65 prisoners 42.

**B:** The names of those who did the right thing, while most just stood by, should not go unmentioned: Avir Kagan, David Leicher and Perry Fersko 41.

**C:** Less biting - although many of the authors have been married for a long time, so we might be misremembering.

**D:** This is, of course, not an issue of absolutes, as one just has to think of the German Mettwurst, Steak tartare or Sushi, where raw meat or seafood still form an integral part of our diet. Although the first two items are far from common. Interestingly, the German biologist Mark Benecke observed that practitioners of sexual cannibalism mimic the prevalent culinary habits of their environment, of all the cannibals he studied only the Japanese Issei Sagawa ate some raw meat (which he compared to tuna), but also preferred to fry most of it 59. However, on a whole wide spread cannibalism for purely nutritional reasons seems unlikely to have been common in hominins as the caloric value of humans is low compared to other food sources available during the Paleolithic 60.

**E:** For comparison: The odds of being killed by lightning have been calculated as 1 in 1.61x105 (http://www.nsc.org/learn/safety-knowledge/Pages/injury-facts-chart.aspx).

**F:** There is an approximately 2-fold variation in the average life expectancy of different mouse strains (which does not correlate with cancer rates), for example the average life span of bred C57BL/6J females is 561 days, of bred AKR/J females 269 days 76. Taking an average life span over several different strains to calculate a relative cancer risk gives a comparable number to that of a wild mouse: 1.38 versus 1.45×10^-8^.

**G:** This seems to be not universally true, as, for example, erythrocytes are bigger in larger animals, even in closely related species of geckos 104. However, using again the calculation put forward by Conlon and Raff, we find that the difference between human and mouse is 2,800-fold with regards to mass and 3,333-fold with regards to cell numbers 84.

**H:** Although the two afflicted individuals concerned were zoo-housed, i.e. compared to their natural habitat lived under conditions of unusually high oxygen concentrations 120.

**I:** Although lymphoma in cats is believed to be often associated with viral infections, such as the Feline leukaemia virus 151.

**J:** More controversially LFS2, associated with a mutation in the CHEK2 gene, and a third variant of LFS have also been suggested (168, 169, respectively).

**K:** An alternative approach would be restoring wild-type TP53 in LFS patients via gene editing, such as CRISPR/Cas or zinc finger nucleases. The latter are currently used in a clinical trial to halt the physical deterioration associated with Hunter Syndrome. However, it should be noted that it has been estimated that only 1% of all liver cells need to be successfully targeted to treat this disease, while with LFS a much higher percentage in (almost) all tissues would need to be corrected. This would make the imprecise addition of multiple copies more feasible than the precise insertion of a single copy per every cell. As with every gene (editing) therapy approach there are also lethal dangers associated with both strategies.

## Figures and Tables

**Table 1:** Mathematical relative cancer risk versus real world observations in various mammalian species

Common name	Relative cancer risk ❶	Cancer rate	Adjusted annual cancer rate per 100,000	% death due to cancer	Comment
Etruscan shrew	1.42983E-10	7.69% 72			V
bumblebee bat	1.31084E-09				
mouse	1.45239E-08				
Brandt's bat	6.4179E-08				
golden hamster	5.6519E-07				
naked mole rat	7.85105E-07	0%		0%	VIII
rat	1.71435E-06				
rabbit	3.81396E-05				
hare	0.000627254				
domestic cat	0.00069518		412 9		I
Tasmanian devil	0.002109824				
dog (Yorkshire terrier)	0.002646611		507 9	27% 108	II, IX, X
roe deer	0.018079254			2% 109	
dog (beagle)	0.034867133		507 9	32.8% 108	II, X
cheetah (female)	0.041421668	23.08% 72			
dog (Labrador retriever)	0.046442134		507 9	31.2% 108	II, X
cheetah (male)	0.059151521	23.08% 72			
dog (Rottweiler)	0.071473291		507 9	45.3% 108	II, X
sheep (ewe)	0.183092961		0.03 9		
tiger (female)	0.293179314	11.76% 72			
sheep (ram)	0.33561928		0.03 9		
tiger (male)	0.462330424	11.76% 72			
California sea lion (female)	0.730512179	5,71% 72			IV, VI
lion (female)	0.733822333	1.92% 72			
human (Asia)	0.809250789				
gorilla (female)	0.89228487				
**human (average)**	**1**	**31% 110**	**476 9**	**14.3% 110**	
lion (male)	1.179427339	1.92% 72			
human (North America)	1.586146251				
gorilla (male)	2.456701544				
polar bear (female)	2.45714029				
California sea lion (male)	4.995623001	5.71% 72			IV, VI
polar bear (male)	10.15662364				
horse	13.43438078		41 9		
cow (female)	18.72013426		75 9		
giraffe (female)	29.64927802				
elephant seal (female)	35.77005876				
cow (bull)	38.21323624		75 9		
giraffe (male)	93.5055589				
beluga whale	125.8347077		570 9	27% 9	III
hippopotamus	133.2367493				
elephant seal (bull)	136.857391				
African elephant (female)	521.9576689	3.11% 72			VII
African elephant (male)	1745.405691	3.11% 72			VII
blue whale	749033.9482				

❶. Relative cancer risk was defined as the product of average weight times (as surrogate for cell number) average life expectancy (as surrogate for cell divisions) times adjustment according to Kleiber's Law. Data was put into relation to the average human. Only extreme sexual dimorphism noted. In the absence of available data information from closest related species was used or adjusted life span was incorporated. **Comments:** I. increased lymphoma risk due to second hand smoke 111. II. Increased lung and nasal cancer risk due to second hand smoke (112, 113, respectively). III. Likely not representative, possible link to environmental contaminants in the St. Lawrence estuary 9. IV. At least some populations increased genetic risk of urogenital carcinoma 101. V. Data from “Treeshrew” used. VI. Data from “Harbor seal” used from 72. VII. Data from “Elephant” was used. VIII. As mentioned two individuals living in captivity, i.e. unusual living conditions, were found to harbour a tumour. IX. No data provided by 108, so canine average is given for “% death due to cancer”. X. Canine average is given for “adjusted annual cancer rate per 100,000” 110. Data for developed countries was used 72; data is given without sex-biased.

**Table A:** Some observations from the veterinary clinical practice

FISS: Feline injection-site (fibro)sarcoma 128-130	Feline injection-site fibrosarcoma are attributable to vaccination and administration of other pharmaceutical products. The tumours can spread along fascial planes and frequently reoccur after surgical removal. Nevertheless, benefits of vaccination clearly outweigh the cancer risks. Development of sarcomas at the site of repeated trauma or foreign body implants could be demonstrated in rats via the injection of food colourings, soya oil, iron derivatives or the implantation of solid materials, including plastic films. These findings were suggested to be due to the induction of chronic inflammation and subsequent cell metaplasia and gave rise to concerns over implants and prostheses used in human medicine. However, available evidence does not suggest these are associated with higher cancer risks. Possible etiologic factors for feline injection-site sarcomas include single nucleotide polymorphisms of feline p53 and upregulation of genes also differentially expressed in human soft-tissue sarcomas like *FAP* (fibroblast activation protein α) and *PRAME* (preferentially expressed antigen in melanoma).
Cancer in budgerigars (*Melopsittacus undulatus*) 131-134	It was observed in veterinary practice that there is a higher cancer rate in budgerigars than in all other ornamental birds. Most frequently recognized where renal and haematological neoplasia. The aetiology for the high susceptibility of budgerigars to neoplasia remains an unsolved mystery, but several lines of reasoning led to the suggestion of a retrovirus as a common causative agent. Nevertheless, investigations could not find evidence of an exogenous, replicating retrovirus using primary cell cultures of kidney tissue from budgerigars with renal neoplasia, amongst other material. However, in the case of myeloblastic myeloid leukosis in budgerigars, the subgroup J avian leukosis virus was found to favour myeloblastosis and myelocytomatosis. The Aves polyomavirus 1 (APV) was frequently observed in young budgerigars to be associated with inflammatory diseases and the common yeast infection macrorhabdiosis (caused by *Macrorhabdus ornithogaster)* provides a possible explanation for increased cancer incidences in those birds as chronic inflammation provides an underlying basis for the development of cancer.
Equine Sarcoids and bovine papillomavirus 135-139	Papillomaviruses were previously thought to be species-specific, but infection to accidental hosts can occur and commonly results in a in a different pathological outcome to that in the normal host. Cattle warts induced by the bovine papillomavirus (BPV) are benign tumours and generally regress without eliciting any serious complications. In equids, including horses, donkeys, mules and zebras the BPV can cause so-called *equine sarcoids. D*espite being classified as benign these sarcoids cause a high morbidity in the equids and not infrequently lead to the decision to euthanize the animal. The tumour commonly occurs on multiple body-locations, rarely regresses and very often recurs after surgical excision. While in the early 20^th^ century inoculation experiments already led to speculations suggesting an infectious agent as causative agent, it remains unclear how the bovine papillomavirus reaches the equids. In cattle, BPV is transmitted by contact between animals or contact with fomites. Speculations in transmission to equids include face flies (*Musca autumnalis*) as potential vectors, infectious cell lines in analogy to canine transmissible venereal tumours or, as BPV1 RNA isolated from equine sarcoids was found to encode a unique deletion, it was even suggested that a novel variant of virus had evolved in equines. So far, no evidence has emerged supporting any hypothesis. Standard treatment includes surgery, chemotherapy and immunotherapy including the off-label use of Bacille de Calmette et Guérin (BCG) vaccination.

**Table 2:** Five most common cancers in adult and paediatric humans and other mammals

Human ①		Other mammals			
Adult	Paediatric	Leporine ①	Canine ③	Feline ④	Equine ⑤
Breast	Leukaemia	Uterine cancer	Mast cell tumours	Skin Cancer	Sarcoids
Prostate	Brain, other CNS and intracranial tumours	Lymphoma	Soft tissue sarcoma	Leukaemia and lymphoma	Squamous cell carcinomas
Lung	Lymphoma	Interstitial cell tumours of the testes	Lymphoma	Mouth and pharynx	Lymphoma
Bowel	Soft tissue sarcoma	Mammary tumours	Osteosarcoma	Stomach and intestine	Melanomas
Melanoma skin cancer	Sympathetic nervous system tumours	Lymphosarcoma (Lymphoma)	Mammary carcinoma	Mammary gland	Granulosa cell tumours

**Sources:** ①. 150. ②. http://www.netvet.co.uk/rabbits/cancer-and-growths.htm. ③. 108. ④. 8, roughly similar to more current data found at http://www.petwave.com/Cats/Health/Cancer.aspx, and http://www.petwave.com/Cats/Health/Cancer.aspx. ⑤. http://www.horseandhound.co.uk/horse-care/vet-advice/the-most-common-cancers-in-horses-312896.

**Table B:** The major histocompatibility complex (MHC)

The major histocompatibility complex (MHC) is a group of genes that code for proteins found on the surfaces of cells that help the immune system recognize foreign substances; more preciously pathogen-derived peptides bound to MHC molecules. In humans, the complex is also referred to human leukocyte antigen (HLA). MHC genes are grouped into class I, class II, and class III depending on their location on the respective chromosome, structure, and function. MHC class I molecules, which are present on all nucleated cells 152, are also functional in the innate immune system by acting as ligands of inhibitory killer cell immunoglobulin-like receptors (KIRs) on natural killer (NK) cells. NK cells play a key role because they have the unique ability to recognize and non-specifically destroy cells lacking self MHC class I molecules. Because all (healthy) nucleated cells express self MHC class I molecules, inhibitory KIRs ensure that NK cells are not attack normal cells but they kill infected and tumour cells, which downregulate MHC molecules 153.

## References

[1] Ferraris ZA, Ferraris VA (1997). The Women of Salerno: Contribution to the Origins of Surgery from Medieval Italy. Ann Thorac Surg.

[2] Naumann DN, Bowley DM, Midwinter MJ, Walker A, Pallister I (2016). High-Fidelity Simulation Model of Pelvic Hemorrhagic Trauma: The Future for Military Surgical Skills Training? Mi. Med.

[3] Jucker M (2010). The benefits and limitations of animal models for translational research in neurodegenerative diseases. Nat Med.

[4] Chong AS, Alegre M-L, Miller ML, Fairchild RL (2013). Lessons and Limits of Mouse Models. Cold Spring Harb Perspect Med.

[5] van Blijswijk J, Schraml BU, Sousa CRe (2013). Advantages and limitations of mouse models to deplete dendritic cells. Eur J Immunol.

[6] Beck JA, Lloyd S, Hafezparast M, Lennon-Pierce M, Eppig JT, Festing MFW (2000). Genealogies of mouse inbred strains. Nat Genet.

[7] Yang H, Wang H, Jaenisch R (2014). Generating genetically modified mice using CRISPR/Cas-mediated genome engineering. Nat Protocols.

[8] Dorn CR (1967). The Epidemiology of Cancer in Animals. Calif Med.

[9] Martineau D, Lemberger K, Dallaire A, Labelle P, Lipscomb TP, Michel P (2002). Cancer in wildlife, a case study: beluga from the St. Lawrence estuary, Quebec, Canada. Environ Health Perspect.

[10] Madsen T, Arnal A, Vittecoq M, Bernex F, Abadie J, Labrut S (2017). Cancer Prevalence and Etiology in Wild and Captive Animals. Ecology and Evolution of Cancer.

[11] Nielsen J, Hedeholm RB, Heinemeier J, Bushnell PG, Christiansen JrS, Olsen J (2016). Eye lens radiocarbon reveals centuries of longevity in the Greenland shark (Somniosus microcephalus). Science.

[12] Weiss RA, Vogt PK (2011). 100 years of Rous sarcoma virus. J Exp Med.

[13] Androutsos G, Karamanou M (2009). Bernard Peyrilhe (1737-1804) and the first experimental transmission of cancer. J Buon.

[14] Sticker A (1906). Infektiöse und krebsige Geschwülste an den äußeren Geschlechtsorganen des Hundes. Arch Klin Chir.

[15] Novinski MA (1876). Zur Frage über die Impfung der Krebsigen Geschwulste. Zentralbl Med Wissensch.

[16] Mukaratirwa S, Gruys E (2003). Canine transmissible venereal tumour: Cytogenetic origin, immunophenotype, and immunobiology. A review. Vet Q.

[17] Murgia C, Pritchard JK, Kim SY, Fassati A, Weiss RA (2006). Clonal Origin and Evolution of a Transmissible Cancer. Cell.

[18] Liao K-W, Hung S-W, Hsiao Y-W, Bennett M, Chu R-M (2003). Canine transmissible venereal tumor cell depletion of B lymphocytes: molecule(s) specifically toxic for B cells. Vet Immunol Immunopathol.

[19] Ganguly B, Das U, Das AK (2016). Canine transmissible venereal tumour: a review. Vet Comp Oncol.

[20] Brindley DC, Banfield WG (1961). A Contagious Tumor of the Hamster. J Natl Cancer Inst.

[21] Banfield WG, Woke PA, Mackay CM, Cooper HL (1965). Mosquito Transmission of a Reticulum Cell Sarcoma of Hamsters. Science.

[22] Weinberger MA, Banfield WG (1965). Fine Structure of a Transplantable Reticulum Cell Sarcoma. I. Light and Electron Microscopy of Viable and Necrotic Tumor Cells. J Natl Cancer Inst.

[23] Ashbel R (1945). Spontaneous Transmissible Tumours in the Syrian Hamster. Nature.

[24] Copper HL, Mackay CM, Banfield WG (1964). Chromosome Studies of a Contagious Reticulum Cell Sarcoma of the Syrian Hamster. J Natl Cancer Inst.

[25] Ostrander EA, Davis BW, Ostrander GK (2016). Transmissible Tumors: Breaking the Cancer Paradigm. Trends Genet.

[26] Metzger MJ, Reinisch C, Sherry J, Goff SP (2015). Horizontal transmission of clonal cancer cells causes leukemia in soft-shell clams. Cell.

[27] Metzger MJ, Villalba A, Carballal MJ, Iglesias D, Sherry J, Reinisch C (2016). Widespread transmission of independent cancer lineages within multiple bivalve species. Nature.

[28] Jones ME, Cockburn A, Hamede R, Hawkins C, Hesterman H, Lachish S (2008). Life-history change in disease-ravaged Tasmanian devil populations. Proc Natl Acad Sci USA.

[29] Stammnitz MR, Coorens THH, Gori KC, Hayes D, Fu B, Wang J (2018). The Origins and Vulnerabilities of Two Transmissible Cancers in Tasmanian Devils. Cancer Cell.

[30] Tovar C, Pye RJ, Kreiss A, Cheng Y, Brown GK, Darby J (2017). Regression of devil facial tumour disease following immunotherapy in immunised Tasmanian devils. Sci Rep.

[31] Epstein B, Jones M, Hamede R, Hendricks S, McCallum H, Murchison EP (2016). Rapid evolutionary response to a transmissible cancer in Tasmanian devils. Nat Commun.

[32] Murchison EP, Tovar C, Hsu A, Bender HS, Kheradpour P, Rebbeck CA (2010). The Tasmanian devil transcriptome reveals Schwann cell origins of a clonally transmissible cancer. Science.

[33] Sunila I, Sunila, I, Farley A (1989). Environmental limits for survival of sarcoma cells from the soft shell clam, Mya arenaria. Dis aquat Org.

[34] Henwood C (2001). The Discovery of the Syrian (Golden) Hamster, Mesocricetus auratus. The Journal of the British Hamster Association.

[35] Brüniche-Olsen A, Jones ME, Austin JJ, Burridge CP, Holland BR (2014). Extensive population decline in the Tasmanian devil predates European settlement and devil facial tumour disease. Biol Lett.

[36] Murchison EP, Wedge DC, Alexandrov LB, Fu B, Martincorena I, Ning Z (2014). Transmissible Dog Cancer Genome Reveals the Origin and History of an Ancient Cell Lineage. Science.

[37] Hornblum AM (1997). They were cheap and available: prisoners as research subjects in twentieth century America. BMJ.

[38] Moore AE, Rhoads CP, Southam CM (1957). Homotransplantation of human cell lines. Science.

[39] Southam CM (1958). Homotransplantation of human cell lines. Bull N Y Acad Med.

[40] Goliszek A (2003). In the Name of Science. New York: St. Martin's Press.

[41] Hornblum A (2013). M. NYC's forgotten cancer scandal. New York: New York Post, 28^th^ December.

[42] Skloot R (2010). The Immortal Life of Henrietta Lacks. New York: Crown Publishing Grouo.

[43] Scanlon EF, Hawkins RA, Fox WW, Smith WS (1965). Fatal Homotransplanted Melanoma: A Case Report. Cancer.

[44] Ehrlich P (1957). The collected papers of Paul Ehrlich. Himmelweit F, editor. II. London: Pergamon Press.

[45] Isoda T, Ford AM, Tomizawa D, van Delft FW, De Castro DG, Mitsuiki N (2009). Immunologically silent cancer clone transmission from mother to offspring. Proc Natl Acad Sci U S A.

[46] Welsh JS (2011). Contagious cancer. Oncologist.

[47] Muehlenbachs A, Bhatnagar J, Agudelo CA, Hidron A, Eberhard ML, Mathison BA (2015). Malignant Transformation of Hymenolepis nana in a Human Host. N Engl J Med.

[48] Conn DB (2016). Correspondence: Malignant Transformation of Hymenolepis nana in a Human Host. N Engl J Med.

[49] Smith GD, Travis L (2011). Getting to Know Human Papillomavirus (HPV) and the HPV Vaccines. J Am Osteopath Assoc.

[50] Yoshimura K (2017). Current status of HIV/AIDS in the ART era. J Infect Chemother.

[51] Unemo M, Bradshaw CS, Hocking JS, de Vries HJC, Francis SC, Mabey D (2017). Sexually transmitted infections: challenges ahead. Lancet Infect Dis.

[52] Groce NE, Trasi R (2004). Rape of individuals with disability: AIDS and the folk belief of virgin cleansing. The Lancet.

[53] Mead S, Stumpf MPH, Whitfield J, Beck JA, Poulter M, Campbell T (2003). Balancing Selection at the Prion Protein Gene Consistent with Prehistoric Kurulike Epidemics. Science.

[54] Soldevila M, Andres AM, Ramirez-Soriano A, Marques-Bonet Tomas, Calafell F, Navarro A (2006). The prion protein gene in humans revisited: Lessons from a worldwide resequencing study. Genome Res.

[55] Mead S, Whitfield J, Poulter M, Shah P, Uphill J, Beck J (2008). Genetic susceptibility, evolution and the kuru epidemic. Philos. Trans. R. Soc. B.

[56] Whitfield JT, Pako WH, Collinge J, Alpers MP (2008). Mortuary rites of the South Fore and kuru. Philos. Trans. R. Soc. B.

[57] Lindenbaum S (2008). Understanding kuru: the contribution of anthropology and medicine. Philos. Trans. R. Soc. B.

[58] Gowlett JAJ (2016). The discovery of fire by humans: a long and convoluted process. Philos. Trans. R. Soc. B.

[59] Benecke M (2007). Mordspuren. Bergisch Gladbach: Gustav Lübbe Verlag.

[60] Cole J (2017). Assessing the calorific significance of episodes of human cannibalism in the Palaeolithic. Sci Rep.

[61] Sharp PM, Hahn BH (2011). Origins of HIV and the AIDS Pandemic. Cold Spring Harb Perspect Med.

[62] Khabbaz RF, Heneine W, George JR, Parekh B, Rowe T, Woods T (1994). Infection of a Laboratory Worker with Simian Immunodeficiency Virus. N Engl J Med.

[63] Penn I, Klein G, Weinhouse S (1978). Tumors Arising in Organ Transplant Recipients. Advances in Cancer Research. 28. Academic Press.

[64] Barozzi P, Luppi M, Facchetti F, Mecucci C, Alu M, Sarid R (2003). Post-transplant Kaposi sarcoma originates from the seeding of donor-derived progenitors. Nat Med.

[65] Gartner HV, Seidl C, Luckenbach C, Schumm G, Seifried E, Ritter H (1996). Genetic analysis of a sarcoma accidentally transplanted from a patient to a surgeon. N Engl J Med.

[66] Gugel E, Sanders M (1986). Needle-Stick Transmission of Human Colonic Adenocarcinoma. N Engl J Med.

[67] Tomasetti C, Vogelstein B (2015). Cancer risk: role of environment-response. Science.

[68] Wu S, Powers S, Zhu W, Hannun YA (2016). Substantial contribution of extrinsic risk factors to cancer development. Nature.

[69] MacNeil MA, McMeans BC, Hussey NE, Vecsei P, Svavarsson J, Kovacs KM (2012). Biology of the Greenland shark Somniosus microcephalus. J Fish Biol.

[70] Nagy JD, Victor EM, Cropper JH (2007). Why don't all whales have cancer? A novel hypothesis resolving Peto's paradox. Integr Comp Biol.

[71] Leroi AM, Koufopanou V, Burt A (2003). Cancer selection. Nat Rev Cancer.

[72] Abegglen LM, Caulin AF, Chan A (2015). Potential mechanisms for cancer resistance in elephants and comparative cellular response to dna damage in humans. JAMA.

[73] Peto R, Roe FJ, Lee PN, Levy L, Clack J (1975). Cancer and ageing in mice and men. Br J Cancer.

[74] Peto R (2016). Epidemiology, multistage models, and short-term mutagenicity tests 1. Int J Epidemiol.

[75] Hoag WG (1963). Spontaneous Cancer in Mice. Ann N Y Acad Sci.

[76] Biology of the Laboratory Mouse, edited by E (1966). L. Green. Dover Publications, Inc. Second edition, New York.

[77] Trotte MNS, Santos BF, Rodrigo M, Tortelly R Spontaneous neoplasms in mice from a center of a laboratory animal breeding. 2010; 827-36 p.

[78] Warburg O (1956). On the Origin of Cancer Cells. Science.

[79] Liberti MV, Locasale JW (2016). The Warburg Effect: How Does it Benefit Cancer Cells?. Trends Biochem Sci.

[80] Vander Heiden MG, Cantley LC, Thompson CB (2009). Understanding the Warburg Effect: The Metabolic Requirements of Cell Proliferation. Science.

[81] Adelman R, Saul RL, Ames BN (1988). Oxidative damage to DNA: relation to species metabolic rate and life span. Proc Natl Acad Sci USA.

[82] Yang Y, Karakhanova S, Werner J, Bazhin AV (2013). Reactive Oxygen Species in Cancer Biology and Anticancer Therapy. Curr Med Chem.

[83] Kleiber M (1932). Body size and metabolism. Hilgardia.

[84] Conlon I, Raff M (1999). Size Control in Animal Development. Cell.

[85] Dang CV (2012). Links between metabolism and cancer. Genes Dev.

[86] Dang CV (2015). A metabolic perspective of Peto's paradox and cancer. Philos. Trans. R. Soc. B.

[87] Hulbert A, Pamplona R, Buffenstein R, Buttemer W (2007). Life and Death: Metabolic Rate, Membrane Composition, and Life Span of Animals. Physiol Rev.

[88] Takemoto K, Ii M, Nishizuka SS (2016). Importance of metabolic rate to the relationship between the number of genes in a functional category and body size in Peto's paradox for cancer. R Soc Open Sci.

[89] West GB, Brown JH, Enquist BJ (1997). A General Model for the Origin of Allometric Scaling Laws in Biology. Science.

[90] Darveau C-A, Suarez RK, Andrews RD, Hochachka PW (2002). Allometric cascade as a unifying principle of body mass effects on metabolism. Nature.

[91] West GB, Savage VM, Gillooly J, Enquist BJ, Woodruff WH, Brown JH (2003). Why does metabolic rate scale with body size?. Nature.

[92] Maciak S, Michalak P (2014). Cell size and cancer: a new solution to Peto's paradox?. Evol Appl.

[93] Costantini D, Smith S, Killen SS, Nielsen J, Steffensen JF (2017). The Greenland shark: A new challenge for the oxidative stress theory of ageing?. Comp Biochem Physiol A Mol Integr Physiol.

[94] Katzourakis A, Magiorkinis G, Lim AG, Gupta S, Belshaw R, Gifford R (2014). Larger Mammalian Body Size Leads to Lower Retroviral Activity. PLOS Pathog.

[95] Nelson PN, Hooley P, Roden D, Davari Ejtehadi H, Rylance P, Warren P (2004). Human endogenous retroviruses: transposable elements with potential?. Clin Exp Immunol.

[96] Belshaw R, Pereira V, Katzourakis A, Talbot G, Paces J, Burt A (2004). Long-term reinfection of the human genome by endogenous retroviruses. Proc Natl Acad Sci USA.

[97] Frame MC (2004). Newest findings on the oldest oncogene; how activated src does it. J Cell Sci.

[98] Gamperl R, Ehmann C, Bachmann K (1982). Genome size and heterochromatin variation in rodents. Genetica.

[99] Du B, Wang D (2006). C-values of Seven Marine Mammal Species Determined by Flow Cytometry. Zoolog Sci.

[100] Prochazka M, Gaskins HR, Shultz LD, Leiter EH (1992). The nonobese diabetic scid mouse: model for spontaneous thymomagenesis associated with immunodeficiency. Proc Natl Acad Sci USA.

[101] Browning HM, Gulland FMD, Hammond JA, Colegrove KM, Hall AJ (2015). Common cancer in a wild animal: the California sea lion (Zalophus californianus) as an emerging model for carcinogenesis. Philos. Trans. R. Soc. B.

[102] Stewart BE, Stewart REA (1989). Delphinapterus leucas. Mammalian Species.

[103] Stewart REA, Campana SE, Jones CM, Stewart BE (2006). Bomb radiocarbon dating calibrates beluga (Delphinapterus leucas) age estimates. Can J Zool.

[104] Starostova Z, KratochviL L, Frynta D (2005). Dwarf and giant geckos from the cellular perspective: the bigger the animal, the bigger its erythrocytes?. Funct Ecol.

[105] Nonnenmacher L, Hasslacher S, Zimmermann J, Karpel-Massler G, La Ferla-Brühl K, Barry SE (2016). Cell Death Induction in Cancer Therapy & minus; Past, Present, and Future. Crit Rev Oncog.

[106] Nagy JD (2004). Competition and natural selection in a mathematical model of cancer. Bull Math Biol.

[107] Nagy JD (2007). Hypertumors in cancer can be caused by tumor phosphorus demand. PAMM.

[108] Dobson JM (2013). Breed-Predispositions to Cancer in Pedigree Dogs. ISRN Vet Sci.

[109] Aguirre AA, Bröjer C, Mörner T (1999). Descriptive Epidemiology of Roe Deer Mortality in Sweden. J Wildl Dis.

[110] Torre LA, Bray F, Siegel RL, Ferlay J, Lortet-Tieulent J, Jemal A (2015). Global cancer statistics, 2012. CA Cancer J Clin.

[111] Bertone-Johnson E, A Snyder L, Moore A (2002). Environmental tobacco smoke and risk of malignant lymphoma in pet cats. Am J Epidemiol.

[112] Reif JS, Dunn K, Ogilvie GK, Harris CK (1992). Passive Smoking and Canine Lung Cancer Risk. Am J Epidemiol.

[113] Reif JS, Bruns C, Lower KS (1998). Cancer of the Nasal Cavity and Paranasal Sinuses and Exposure to Environmental Tobacco Smoke in Pet Dogs. Am J Epidemiol.

[114] Hanahan D, Weinberg RA (2000). The hallmarks of cancer. Cell.

[115] Hanahan D, Weinberg RA (2011). Hallmarks of cancer: the next generation. Cell.

[116] Joerger AC, Fersht AR (2016). The p53 Pathway: Origins, Inactivation in Cancer, and Emerging Therapeutic Approaches. Annu Rev Biochem.

[117] Vazquez JM, Sulak M, Chigurupati S, Lynch VJ (2018). A Zombie LIF Gene in Elephants Is Upregulated by TP53 to Induce Apoptosis in Response to DNA Damage. Cell Rep.

[118] Kim EB, Fang X, Fushan AA, Huang Z, Lobanov AV, Han L (2011). Genome sequencing reveals insights into physiology and longevity of the naked mole rat. Nature.

[119] Tian X, Azpurua J, Hine C, Vaidya A, Myakishev-Rempel M, Ablaeva J (2013). High-molecular-mass hyaluronan mediates the cancer resistance of the naked mole rat. Nature.

[120] Delaney MA, Ward JM, Walsh TF, Chinnadurai SK, Kerns K, Kinsel MJ (2016). Initial Case Reports of Cancer in Naked Mole-rats (Heterocephalus glaber). Vet Pathol.

[121] Deuker M.M, Lewis K.N, Ingaramo M, Kimmel J, Buffenstein R, Settleman J (2020). Unprovoked Stabilization and Nuclear Accumulation of the Naked Mole-Rat p53 Protein. Sci Rep.

[122] Seluanov A, Hine C, Azpurua J, Feigenson M, Bozzella M, Mao Z (2009). Hypersensitivity to contact inhibition provides a clue to cancer resistance of naked mole-rat. Proc Natl Acad Sci USA.

[123] Shepard A, Kissil J.L (2020). The use of non-traditional models in the study of cancer resistance - the case of the naked mole rat. Oncogene.

[124] Tian X, Azpurua J, Ke Z, Augereau A, Zhang ZD, Vijg J (2015). INK4 locus of the tumor-resistant rodent, the naked mole rat, expresses a functional p15/p16 hybrid isoform. Proc Natl Acad Sci USA.

[125] Vogelstein B, Kinzler KW (1993). The multistep nature of cancer. Trends Genet.

[126] Azpurua J, Ke Z, Chen IX, Zhang Q, Ermolenko DN, Zhang ZD (2013). Naked mole-rat has increased translational fidelity compared with the mouse, as well as a unique 28S ribosomal RNA cleavage. Proc Natl Acad Sci USA.

[127] Seluanov A, Gladyshev VN, Vijg J, Gorbunova V (2018). Mechanisms of cancer resistance in long-lived mammals. Nat Rev Cancer.

[128] Mucha D, Laberke S, Meyer S, Hirschberger J (2014). Lack of association between p53 SNP and FISS in a cat population from Germany. Vet Comp Oncol.

[129] Wei Q., Ramsey S.A, Larson M.K, Berlow N.E, Ochola D, Shiprack C, Kashyap A, Seguin B, Keller C, Löhr C.V (2019). Elucidating the transcriptional program of feline injection-site sarcoma using a cross-species mRNA-sequencing approach. BMC Cancer.

[130] Woodward K.N (2011). Origins of Injection-Site Sarcomas in Cats: The Possible Role of Chronic Inflammation - A Review. ISRN Vet Sci.

[131] Khordadmehr M, Ashrafi-Helana J, Madadi M.S, Jarolmasjed S.H (2015). Natural Unusual Myeloblastosis in a Budgerigar (Melopsittacus undulatus): Histopathologic Diagnosis. Avian Dis.

[132] Ma J, Wu R, Tian Y, Zhang M, Wang W, Li Y, Tian F, Cheng Y, Yan Y, Sun J (2019). Isolation and characterization of an Aves polyomavirus 1 from diseased budgerigars in China. Vet Microbiol.

[133] Powers L.V, Mitchell M.A, Garner M.M (2019). Macrorhabdus ornithogaster Infection and Spontaneous Proventricular Adenocarcinoma in Budgerigars (Melopsittacus undulatus). Vet Pathol.

[134] Simova-Curd S.A, Huder J.B, Boeni J, Robert N, Hatt J.M (2010). Investigations on the diagnosis and retroviral aetiology of renal neoplasia in budgerigars (Melopsittacus undulatus). Avian Pathol.

[135] Bogaert L, Woodham A.W, Da Silva D.M, Martens A, Meyer E, Kast W.M (2015). A novel murine model for evaluating bovine papillomavirus prophylactics/therapeutics for equine sarcoid-like tumours. J Gen Virol.

[136] Chambers G, Ellsmore V.A, apos Brien, P.M Reid, S.W.J Love, S Campo, M.S Nasir, L (2003). Association of bovine papillomavirus with the equine sarcoid. J Gen Virol.

[137] Lunardi M, de Alcantara B.K, Otonel R.A.A, Rodrigues W.B, Alfieri A.F, Alfieri A.A (2013). Bovine papillomavirus type 13 DNA in equine sarcoids. J Clin Microbiol.

[138] Nasir L, Campo M.S (2008). Bovine papillomaviruses: their role in the aetiology of cutaneous tumours of bovids and equids. Vet Dermatol.

[139] Wilson A.D, Armstrong E.L.R, Gofton R.G, Mason J, De Toit N, Day M.J (2013). Characterisation of early and late bovine papillomavirus protein expression in equine sarcoids. Vet Microbiol.

[140] Hammond AS, Royer DF, Fleagle JG (2017). The Omo-Kibish I pelvis. J Hum Evol.

[141] Odes E, Randolph-Quinney P, Steyn M, Throckmorton Z, Smilg J, Zipfel B, et al. Earliest hominin cancer: 1.7-million-year old osteosarcoma from Swartkrans Cave, South Africa. S Afr J Sci. 2016; 112(7/8): #2015-0471 p

[142] Randolph-Quinney PS, Williams SA, Steyn M, Meyer MR, Smilg JS, Churchill SE Osteogenic tumour in Australopithecus sediba: Earliest hominin evidence for neoplastic disease S Afr J Sci. 2016; 112: 1-7.

[143] Jones DS, Podolsky SH, Greene JA (2012). The Burden of Disease and the Changing Task of Medicine. N Engl J Med.

[144] Schwartz AG, Cote ML (2016). Epidemiology of Lung Cancer. Adv Exp Med Biol.

[145] Debatin KM (2017). Cell death: From initial concepts to pathways to clinical applications - Personal reflections of a clinical researcher. Biochem Biophys Res Commun.

[146] Mora C, Tittensor D.P, Adl S, Simpson A.G.B, Worm B (2011). How Many Species Are There on Earth and in the Ocean?. PLoS Biol.

[147] Ma X, Liu Y, Liu Y, Alexandrov LB, Edmonson MN, Gawad C (2018). Pan-cancer genome and transcriptome analyses of 1,699 paediatric leukaemias and solid tumours. Nature.

[148] Gröbner SN, Worst BC, Weischenfeldt J, Buchhalter I, Kleinheinz K, Rudneva VA (2018). The landscape of genomic alterations across childhood cancers. Nature.

[149] Burdach SEG, Westhoff MA, Steinhauser MF, Debatin KM (2018). Precision medicine in pediatric oncology. Mol Cell Pediatr.

[150] Westhoff M-A, Marschall N, Grunert M, Karpel-Massler G, Burdach S, Debatin K-M (2018). Cell death-based treatment of childhood cancer. Cell Death Dis.

[151] Hartmann K (2012). Clinical Aspects of Feline Retroviruses: A Review. Viruses.

[152] Joffre OP, Segura E, Savina A, Amigorena S (2012). Cross-presentation by dendritic cells. Nat Rev Immunol.

[153] Thielens A, Vivier E, Romagne F (2012). NK cell MHC class I specific receptors (KIR): from biology to clinical intervention. Curr Opin Immunol.

[154] Wissing KM, Fomegne G, Broeders N, Ghisdal L, Hoang AD, Mikhalski D (2008). HLA mismatches remain risk factors for acute kidney allograft rejection in patients receiving quadruple immunosuppression with anti-interleukin-2 receptor antibodies. Transplantation.

[155] Ferrara JL, Levine JE, Reddy P, Holler E (2009). Graft-versus-host disease. The Lancet.

[156] Aiuti A, Naldini L (2016). Safer conditioning for blood stem cell transplants. Nat Biotechnol.

[157] Baker KS, Todd ED, Linda JB, Norma KCR, Joseph PN, Leslie LR (2003). New Malignancies After Blood or Marrow Stem-Cell Transplantation in Children and Adults: Incidence and Risk Factors. J Clin Oncol.

[158] Newell KA, Alonso EM, Whitington PF, Bruce DS, Millis JM, Piper JB (1996). Posttransplant Lymphoproloferative Disease in Pediatric Liver Transplantation: Interplay Between Primary Epstein-Barr Virus Infection and Immunosuppression. Transplantation.

[159] Shapiro RS, McClain K, Frizzera G, Gajl-Peczalska KJ, Kersey JH, Blazar BR (1988). Epstein-Barr virus associated B cell lymphoproliferative disorders following bone marrow transplantation. Blood.

[160] Gross TG, Steinbuch M, DeFor T, Shapiro RS, McGlave P, Ramsay NKC (1999). B cell lymphoproliferative disorders following hematopoietic stem cell transplantation: risk factors, treatment and outcome. Bone Marrow Transplantation.

[161] Gutierrez-Dalmau A, Campistol JM (2007). Immunosuppressive therapy and malignancy in organ transplant recipients: a systematic review. Drugs.

[162] Chinen J, Buckley RH (2010). Transplantation immunology: solid organ and bone marrow. J Allergy Clin Immunol.

[163] Ducasse H, Ujvari B, Solary E, Vittecoq M, Arnal A, Bernex F (2015). Can Peto's paradox be used as the null hypothesis to identify the role of evolution in natural resistance to cancer? A critical review. BMC Cancer.

[164] Rankin KS, Frankel D (2016). Hyaluronan in cancer - from the naked mole rat to nanoparticle therapy. Soft Matter.

[165] Lalloo F, Varley J, Ellis D, Moran A, O'Dair L, Pharoah P (2003). Prediction of pathogenic mutations in patients with early-onset breast cancer by family history. The Lancet.

[166] Gonzalez KD, Noltner KA, Buzin CH, Gu D, Wen-Fong CY, Nguyen VQ (2009). Beyond Li Fraumeni Syndrome: Clinical Characteristics of Families With p53 Germline Mutations. J Clin Oncol.

[167] Malkin D, Li FP, Strong LC, Fraumeni JF, Nelson CE, Kim DH (1990). Germ line p53 mutations in a familial syndrome of breast cancer, sarcomas, and other neoplasms. Science.

[168] Bell DW, Varley JM, Szydlo TE, Kang DH, Wahrer DCR, Shannon KE (1999). Heterozygous Germ Line hCHK2 Mutations in Li-Fraumeni Syndrome. Science.

[169] Bachinski LL, Olufemi S-E, Zhou X, Wu C-C, Yip L, Shete S (2005). Genetic Mapping of a Third Li-Fraumeni Syndrome Predisposition Locus to Human Chromosome 1q23. Cancer Res.

[170] Tyner SD, Venkatachalam S, Choi J, Jones S, Ghebranious N, Igelmann H (2002). p53 mutant mice that display early ageing-associated phenotypes. Nature.

[171] Ferbeyre G, Lowe SW (2002). The price of tumour suppression?. Nature.

[172] Wiese RJ, Willis K (2004). Calculation of longevity and life expectancy in captive elephants. Zoo Biol.

[173] Bloom HJ, Wallace EN, Henk JM (1969). The treatment and prognosis of medulloblastoma in children. A study of 82 verified cases. Am J Roentgenol Radium Ther Nucl Med.

[174] Merchant TE, Conklin HM, Wu S, Lustig RH, Xiong X (2009). Late Effects of Conformal Radiation Therapy for Pediatric Patients With Low-Grade Glioma: Prospective Evaluation of Cognitive, Endocrine, and Hearing Deficits. J Clin Oncol.

[175] Clement SC, Schouten-van Meeteren AY, Boot AM, Claahsen-van der Grinten HL, Granzen B, Sen Han K (2016). Prevalence and Risk Factors of Early Endocrine Disorders in Childhood Brain Tumor Survivors: A Nationwide, Multicenter Study. J Clin Oncol.

[176] Cardous-Ubbink MC, Heinen RC, Bakker PJM, van den Berg H, Oldenburger F, Caron HN (2007). Risk of second malignancies in long-term survivors of childhood cancer. Eur J Cancer.

[177] Rigano KS, Gehring JL, Evans Hutzenbiler BD, Chen AV, Nelson OL, Vella CA (2017). Life in the fat lane: seasonal regulation of insulin sensitivity, food intake, and adipose biology in brown bears. J Comp Physiol B.

[178] Keane M, Semeiks J, Webb Andrew E, Li Yang I, Quesada V, Craig T (2015). Insights into the Evolution of Longevity from the Bowhead Whale Genome. Cell Rep.

[179] Cannon B, Nedergaard J (2004). Brown adipose tissue: function and physiological significance. Physiol Rev.

[180] Hughes DA, Jastroch M, Stoneking M, Klingenspor M (2009). Molecular evolution of UCP1 and the evolutionary history of mammalian non-shivering thermogenesis. BMC Evol Biol.

[181] Leslie M (2018). Small but mighty. Science.

[182] Rasmussen SGF, DeVree BT, Zou Y, Kruse AC, Chung KY, Kobilka TS (2011). Crystal structure of the Beta2 adrenergic receptor-Gs protein complex. Nature.

[183] Keller J.M, Balazs G.H, Nilsen F, Rice M, Work T.M, Jensen B.A (2014). Investigating the Potential Role of Persistent Organic Pollutants in Hawaiian Green Sea Turtle Fibropapillomatosis. Environ Sci Technol.

[184] Hernandez I.B, Kromhout J.Z, Teske E, Hennink W.E, van Nimwegen S.A, Oliveira S (2021). Molecular targets for anticancer therapies in companion animals and humans: what can we learn from each other?. Theranostics.

[185] Fernandez Robledo JA, Yadavalli R, Allam B, Pales Espinosa E, Gerdol M, Greco S, Stevick RJ, Gomez-Chiarri M, Zhang Y, Heil CA (2019). From the raw bar to the bench: Bivalves as models for human health. Dev Comp Immunol.

